# Young Sca-1^+^ bone marrow stem cell-derived exosomes preserve visual function via the miR-150-5p/MEKK3/JNK/c-Jun pathway to reduce M1 microglial polarization

**DOI:** 10.1186/s12951-023-01944-w

**Published:** 2023-06-15

**Authors:** Yuan Wang, Wan-yun Qin, Qi Wang, Xin-na Liu, Xiang-hui Li, Xin-qi Ye, Ying Bai, Yan Zhang, Pan Liu, Xin-lin Wang, Yu-hang Zhou, Hui-ping Yuan, Zheng-bo Shao

**Affiliations:** 1 Department of Ophthalmology, The Second Affiliated Hospital of Harbin Medical University, Harbin, China; 2 Future Medical Laboratory, The Second Affiliated Hospital of Harbin Medical University, Harbin, China; 3 The Key Laboratory of Myocardial Ischemia, Harbin Medical University, Ministry Education, Harbin, China

**Keywords:** Exosomes, Bone marrow Sca-1^+^ cell, Ischemia/reperfusion injury, miR-150-5p, MEKK3

## Abstract

**Background:**

Polarization of microglia, the resident retinal immune cells, plays important roles in mediating both injury and repair responses post-retinal ischemia–reperfusion (I/R) injury, which is one of the main pathological mechanisms behind ganglion cell apoptosis. Aging could perturb microglial balances, resulting in lowered post-I/R retinal repair. Young bone marrow (BM) stem cell antigen 1-positive (Sca-1^+^) cells have been demonstrated to have higher reparative capabilities post-I/R retinal injury when transplanted into old mice, where they were able to home and differentiate into retinal microglia.

**Methods:**

Exosomes were enriched from young Sca-1^+^ or Sca-1^−^ cells, and injected into the vitreous humor of old mice post-retinal I/R. Bioinformatics analyses, including miRNA sequencing, was used to analyze exosome contents, which was confirmed by RT-qPCR. Western blot was then performed to examine expression levels of inflammatory factors and underlying signaling pathway proteins, while immunofluorescence staining was used to examine the extent of pro-inflammatory M1 microglial polarization. Fluoro-Gold labelling was then utilized to identify viable ganglion cells, while H&E staining was used to examine retinal morphology post-I/R and exosome treatment.

**Results:**

Sca-1^+^ exosome-injected mice yielded better visual functional preservation and lowered inflammatory factors, compared to Sca-1^−^, at days 1, 3, and 7 days post-I/R. miRNA sequencing found that Sca-1^+^ exosomes had higher miR-150-5p levels, compared to Sca-1^−^ exosomes, which was confirmed by RT-qPCR. Mechanistic analysis found that miR-150-5p from Sca-1^+^ exosomes repressed the mitogen-activated protein kinase kinase kinase 3 (MEKK3)/JNK/c-Jun axis, leading to IL-6 and TNF-α downregulation, and subsequently reduced microglial polarization, all of which contributes to reduced ganglion cell apoptosis and preservation of proper retinal morphology.

**Conclusion:**

This study elucidates a potential new therapeutic approach for neuroprotection against I/R injury, via delivering miR-150-5p-enriched Sca-1^+^ exosomes, which targets the miR-150-5p/MEKK3/JNK/c-Jun axis, thereby serving as a cell-free remedy for treating retinal I/R injury and preserving visual functioning.

**Supplementary Information:**

The online version contains supplementary material available at 10.1186/s12951-023-01944-w.

## Introduction

Ischemia and reperfusion (I/R)-elicited tissue injuries contributes to morbidity and mortality for a wide variety of diseases. In the case of the retina, I/R injury, which could stem from high intraocular pressure (IOP), results in neural apoptosis [1], and subsequently vision loss, possibly to the point of blindness [2]. One key factor leading to neuron death in I/R is neuroinflammation induced by microglial activation [3, 4]. Indeed, microglia have been observed to be involved in cellular reactions associated with high IOP-caused optic neuropathy. In particular, M1 microglia have been defined as the pro-inflammatory type, producing inflammatory cytokines, such as interleukin (IL)-6, tumor necrosis factor (TNF)-α, and IL-1β in the retina, all of which may contribute to neuronal apoptosis and eventual neuro-destruction [5, 6]. Therefore, new effective treatments for I/R-induced inflammation are required to counteract against these pathological effects. I/R injury increases retinal microglia polarization towards the M1 type, and aging aggravates this polarization tendency, along with being associated with decreased microglial functioning [7, 8]. There, targeting M1 microglia could serve as a therapeutic approach for alleviating retinal I/R-induced neurotoxicity in aged populations.

In our previous study [9], we discovered that when young bone marrow (BM) stem cell antigen-1 positive (Sca-1^+^) cells were transplanted into older mice, these stem cells were able to find their way to the retina, where they differentiated into microglia. Furthermore, these cells were able to reduce cellular apoptosis and death, after acute I/R injury, by activating the fibroblast growth factor 2/Akt signaling pathway. However, the necessity of cell transplantation for current stem cell therapies has several limitations [10], such as concerns over safety and low cell survival efficiency, due to the transplantation process itself and immunological rejection, which have become significant concerns during clinical trials [11]. Therefore, the development of an effective cell-free treatment, to provide anti-inflammatory and micro-environmental protection, with no immune rejection risk, is of great importance. Due to the paracrine activities of stem cells [12], BM cell-derived products, such as exosomes, are capable of acting as a cell-free anti-inflammatory therapy, with low immunogenicity [13].

Exosomes are virus-size membranous vesicles, originating from the endocytic compartment of cells, and range from 30 to 150 nm in diameter. A growing body of evidence indicates that they play a major role in intercellular communication in both physiological and pathological conditions [14]. Exosomes are enriched in mRNAs, miRNAs, other non-coding (nc) RNAs, proteins, and biological factors [15], allowing them to exert paracrine effects on other cells. All of these molecules within exosomes have resulted in these entities becoming a potential delivery method for treating retinal diseases, such as optic nerve crush, glaucoma, laser injury, diabetic retinopathy, etc. [16]. In particular, Xue et al. found that exosome-mediated delivery of an anti-angiogenic peptide, KV11, was more effective in counteracting against pathological angiogenesis, compared to injecting KV11 alone [17]. Additionally, Mead et al. determined that miRNAs within mesenchymal stem cell-derived exosomes were able to maintain retinal ganglion cell survival in a rat optic nerve crush model [18]. Similar observations, using extracellular matrix-localized nanovesicles, were found in a rat model, with retinal ischemia induced by severe intraocular pressure [19]. However, possible therapeutic applications of exosomes for treating I/R-related retinal injuries has not been fully examined. Exosome-associated miRNA has been reported to be more stable and resistant to RNase enzymatic activity, compared to non-exosomal miRNAs [20, 21]. miRNAs, in turn, regulate expression of multiple target genes by binding to mRNAs and inhibiting their translation, or inducing mRNA degradation [22]. In this study, we enriched exosomes from young BM Sca-1^+^ and Sca-1^−^ cells and injected them into the retinas of old mice post-I/R injury. We found that Sca-1^+^-derived exosomes were able to reduce ganglion cell apoptosis, as well as preserve visual function, owing to them being enriched for the miRNA miR-150-5p, which has previously been found to have altered expression levels during neurodegeneration [23]. This exosomal miR-150-5p was able to reduce microglial polarization via suppressing the mitogen-activated protein kinase kinase kinase 3 (MEKK3)/JNK/c-Jun axis, leading to downregulation of pro-inflammatory cytokines, thus demonstrating neuroprotective and anti-inflammatory effects against retinal I/R injury.

## Materials and methods

### Obtaining BM Sca-1^+^ stem cells and culturing

All animal experiments were approved by the Institutional Animal Care and Use Committee of Harbin Medical University, and were carried out in accordance with the Statement for the Use of Animals in Ophthalmic and Vision Research by the Association for Research in Vision and Ophthalmology, as well as the Guide for the Care and Use of Laboratory Animals from the National Institutes of Health. Femurs and tibias from wild-type C57BL/6 mice [2–3 months, totally 90 mice] were flushed with phosphate buffered saline (PBS) to obtain nucleated BM stem cells. These cells were then sorted into 2 categories, Sca-1^+^ or Sca-1^−^ stem cells [24], using a magnetic affinity cell sorting kit, in line with the manufacturer’s instructions (Stem Cell Technology, Canada). After sorting, stem cells were cultured in Iscove's Modified Dulbecco's Medium (Biosharp, China), with 10% (v/v) exosome-depleted fetal bovine serum (FBS; SBI, USA) and 1% antibiotic–antimycotic solution (Beyotime, China), and incubated for 48 h in an incubator, at 37 °C and with 5% CO_2_, for exosome enrichment.

### Isolation and characterization of BM stem cell-derived exosomes

After 48 h culture, cell supernatants were obtained via centrifugation at 5000×*g* for 15 min, filtered through 0.45 μm filters, and concentrated by passing them through 100K ultrafiltration tubes (Millipore, USA). Exosome precipitation and purification was performed using Exo-spin™ exosome size-exclusion columns (Cell GS, UK). Their sizes and shapes were examined using transmission electron microscopy (TEM) (Hitachi, HT-7700, Japan). Nanoparticle tracking analysis (NTA) was used to measure exosome diameter and quantities (NanoFCM, N30E, China). Specific exosome markers cluster of differentiation (CD) 9 (1:1000, Cat # 92726, Abcam), CD81 (1:1000, Cat # ET1611-87, Huabio) and CD63 (1:1000, Cat # 217345, Abcam) [25], were detected with Western blot.

### Establishing the retinal I/R injury mouse model and intravitreal injection of exosomes

To establish the retinal I/R injury animal model, old mice (total 48; 18–20 months) were anaesthetized with 5% chloral hydrate, and both eyes were subjected to retinal I/R injury, as previously described [26]. Briefly, a 500 mL IV bottle, containing sterile salt solution, comprised the normal saline reservoir. This bottle was connected to a 32-gauge needle, and the needle was inserted into the anterior chamber of mouse eyes. The reservoir was then hung from an IV pole extension, and elevated at a height of 1.5 m, resulting in the mouse eye being subjected to 110 mmHg of hydrostatic pressure. The eye was then continuously infused with saline for 1 h, after which the needle was removed to allow for the reperfusion of retinal vasculature, resulting in induction of retinal I/R injury. Mice who received retinal I/R in both eyes were randomly assigned into the following groups: normal control without I/R (Normal), I/R, I/R + Sca-1^+^, and I/R + Sca-1^−^; both eyes were each injected intravitreally with 2 μL of exosomes right after I/R injury.

### Visual function detection

To assess visual function, we selected mice who had a clear refractive medium in both eyes, following modeling and intravitreal injection. Light/dark box exploration and optomotor response tasks were used, as previously described [9]. For light/dark box exploration, briefly, after 2 h of dark adaptation in the dark box, mice (total 9) were placed into the light box (Fig. 2A), and visual function was evaluated, based on data collected for 10 min, in terms of time spent in the light box, as well as the number of transitions between the dark and light boxes via passing through the doorway between them. For the optomotor response task, the mouse was placed on a platform in the light box, and revolving vertical stripes, at 3 different frequencies, were projected on the surrounding LED screen (Fig. 2D–E); the number of mouse head movements elicited by the vertical stripe rotation, for 5 min, was recorded for each frequency. Visual acuity was based on the highest spatial frequency eliciting this optomotor response. Each mouse was tested 3 times per trial, and the data were averaged for further analysis. Three animals from each experimental group were tested.

### Retinal thickness measurements

Histological analysis was used to measure total retinal thickness, as well as for the 5 retinal layers. Mice were anesthetized at 7 days post-retinal I/R, and received trans-cardiac perfusions of 4% paraformaldehyde (PFA). Eyeballs were removed and fixed in 4% PFA overnight at 4 °C, after limbal paracentesis with a sterile 32 G syringe. Fixed eyeballs were dehydrated using increasing percentages of ethanol, from 50, to 70, to 95, to 100%, then paraffin-embedded and sectioned into 4 μm sections. The sections were stained using a hematoxylin and eosin (H&E)-staining kit, following the manufacturer’s instructions (Beyotime, China). All retinal thickness measurements were performed 2 mm away from the optic disc edge.

### Terminal deoxynucleotidyl transferase dUTP nick end labeling (TUNEL) assay and immunofluorescence staining

For the TUNEL assay, mouse eyeballs were harvested at 3 days post-retinal I/R, while they were harvested at 7 days post-injury for immunofluorescence staining. This difference in timepoints is due to the different phases of the I/R injury response [27], in which neuronal apoptosis occurs in the immediate aftermath of the injury, followed by inflammatory reactions, involving immune cell infiltration, as well as subsequent differentiation and polarization into microglia for the clearance of apoptotic neurons. Eyeballs were fixed overnight in 4% PFA at 4 °C, after limbal paracentesis with a sterile 32 G syringe. Subsequently, they were dehydrated using increasing percentages of sucrose, starting at 10%, then 20%, for 2 h each, and finally at 30% for 30 min, all at 4 °C. Eyeballs were then embedded in OCT compound (Sakura Finetek, Japan), and 4 µm transverse sections through the optic disc of the eye were obtained. The resulting tissue sections were fixed with 4% PFA for 20 min, permeabilized with 0.1% Triton X-100 in PBS for 10 min, and blocked with 10% goat serum in PBS for 2 h. For TUNEL staining, the TUNEL assay kit was used (Roche, Switzerland), following the manufacturer’s instructions.

As for immunofluorescence staining, the sections were incubated overnight, at 4 °C, with the following antibodies: NeuN (1:50; Cat # ab177487, Abcam), CD16/CD32 (1:50; Cat # ab223200, Abcam), and ionized calcium-binding adapter molecule 1 (Iba-1, 1:50; Cat # ab283319, Abcam). Sections were then incubated with fluorescein (FITC) AffiniPure goat anti-rabbit (1:200; 111-095-003, Jackson) and Red-X-AffiniPure goat anti-mouse immunoglobulin G (IgG) antibodies (1:200; 115-295-003, Jackson). Nuclei were stained using 4′,6-diamidino-2-phenylindole (DAPI; Beyotime, China) at room temperature for 3 min. Slides were mounted using an anti-fade fluorescence mounting medium (Dako; S3023, Denmark), and fluorescence images were obtained via fluorescence microscopy (Leica, Germany). Quantifications of fluorescence intensity, as well as NeuN^+^ and CD16/CD32^+^ microglia, were performed using ImageJ, for 3 mice from each treatment group.

Retinal flatmounts were carried out by first harvesting and incubating mouse eyeballs for 2 h at 4 °C, followed by dissection of their retinas. Retinas were then placed in 2% Triton X-100, diluted in PBS, for 40 min at − 80 °C, and transferred into blocking buffer (5% normal goat serum in 2% Triton X-100), to be incubated for 2 h at room temperature. Afterwards, they were incubated with Iba-1 and CD16/CD32 primary antibodies, diluted in 2% blocking buffer, overnight at 4 °C, followed by incubation with fluorescein (FITC) AffiniPure goat anti-rabbit (1:200; 111-095-003, Jackson) and Red-X-AffiniPure goat anti-mouse immunoglobulin G (IgG) antibodies (1:200; 115-295-003, Jackson) at room temperature for 2 h. Nuclei were stained using 4′,6-diamidino-2-phenylindole (DAPI; Beyotime, China) at room temperature for 3 min. Retinal flatmounts were then formed by placing the retinas on the slides, vitreous body-side down; the retinas were flattened by applying 4 symmetrical radial incisions, centered on the optic disc, and cover-slipped with Dako fluorescence mounting medium. CD16/CD32^+^ microglia were quantified, using ImageJ, in a blinded fashion, within a 0.01 mm^2^ (100 × 100 μm) rectangular region, from the peripheral edge of the retina; 3 regions from each retinal sample were used.

### BV2 cell culture and uptake of exosomes

BV2, an immortalized murine microglial cell line, was maintained in Dulbecco’s minimal essential medium, supplemented with 10% (v/v) FBS and penicillin/streptomycin (100 units and 100 μg/mL) at 37 °C in a 5% CO_2_ incubator. To establish the lipopolysaccharide (LPS)-induced inflammatory cell model, BV2 at a density of 5 × 10^4^ cells/well were seeded into 6 well plates, cultured overnight, then co-cultured with 100 μg/mL of exomes for 12 h, and exposed to 1 μg/mL LPS (Sigma, USA) for 24 h.

To measure exosome uptake, BV2 cells were seeded at a density of 5 × 10^3^ cells/well into a 24-well chamber slide and cultured overnight. Cells were then co-cultured for 2 h with 10 μM of DiI-labeled exosomes (DiI, Beyotime, China), fixed with 4% PFA for 20 min, permeabilized with 0.1% Triton X-100 in PBS for 10 min, and blocked with 10% goat serum in PBS for 2 h. Afterwards, cells were incubated overnight with primary antibodies against Iba-1 at 4 °C, then incubated with FITC AffiniPure goat anti-rabbit IgG secondary antibody for 60 min in darkness at room temperature. Nuclei were stained with DAPI, and cells imaged by fluorescence microscopy.

### Reverse transcription quantitative real-time PCR (RT-qPCR)

Total RNA from retinas, as well as from BV2 cells, representing microglia, were extracted using TRIzol^®^ Reagent (CWBIO, China), then reverse transcribed into cDNA using the Transcriptor First Strand cDNA Synthesis Kit, in accordance with the manufacturer’s instructions (Roche, Switzerland). qPCR was conducted using NCSYB GREEN qPCR Master Mix (NCBIOTECH, China). As for exosomes, total RNA was extracted using the RNAsimple Total RNA Kit (Tiangen, DP419, China), reverse transcribed into cDNA using the miRcute Plus miRNA First-Strand cDNA Kit (Tiangen, KR211, China), and qPCR performed using the miRcute Plus miRNA qPCR Kit (SYBR Green; Tiangen, FP411, China). Primer sequences used were listed in Additional file 1: Table S1.

### Western blot

Radioimmunoprecipitation assay lysis buffer (Beyotime, China) was used to lyse retinas obtained after removing the lens and anterior portions of mouse eyes, as well as exosomes, plus Sca-1^+^, Sca-1^−^, and BV2 cells, for protein extraction. Proteins were quantified using the BCA Protein Assay Kit (Solarbio, China), and 15 µg of total protein was separated on SDS-PAGE gels (Epizyme, China), then transferred to polyvinylidene difluoride membranes (Millipore, Germany). Primary antibodies used included Sca-1 (1:1000, Cat # 0804-10, HUABIO), IL-6 (1:1000, Cat # 500286, ZENBIO), TNF-α (1:1000, Cat # 346654, ZENBIO), MEKK3 (1:1000, Cat # 381471, ZENBIO), phospho-SAPK (p-SAPK)/JNK (1:1000, Cat # 4668, CST), JNK (1:1000, Cat # 10023, Proteintech), p–c-Jun (1:1000, Cat # R22955, ZENBIO), c-Jun (1:1000, Cat # R23335, ZENBIO), and GAPDH (1:10000, Cat # 10494-1-AP, Proteintech), and protein levels were quantified by ImageJ.

### Constructing exosomal RNA libraries and high-throughput sequencing

Total RNA from exosomes were extracted using TRIzol (Invitrogen, CA, USA), treated with DNase I (Takara, Kusatsu, Japan) to remove any contaminating DNA, and monitored for degradation and contamination with 1% agarose gel electrophoresis. RNA concentration and purity was measured, and 1 µg of total RNA for each sample was used to generate sequencing libraries, using the NEBNext® Multiplex Small RNA Library Prep Set for Illumina^®^ (NEB, USA). The libraries were then sequenced using Illumina Novaseq 6000, and 50 bp single-end reads were generated. Quality control was conducted on raw reads to obtain clean reads for differential expression analysis of miRNA between different samples, using DEseq2 software (P value< 0.05, |log_2_foldchange|> 1).

### Bioinformatics analyses

Target genes for differentially-expressed miRNAs were predicted using miRDB (http://www.mirdb.org/), miRWalk (http://mirwalk.umm.uni-heidelberg.de/), microT (http://diana.cslab.ece.ntua.gr/microT/), miRanda (http://www.microrna.org/microrna/home.do), and TargetScan databases (http://www.targetscan.org/). Gene Ontology (GO) functional annotation and Kyoto Encyclopedia of Genes and Genomes (KEGG) pathway enrichment analyses were conducted using the Database for Annotation, Visualization and Integrated Discovery (http://david.niaid.nih.gov).

### Retrograde labeling of retinal ganglionic cells and quantification

Retrograde labeling of retinal ganglion cells was conducted 3 days prior to retinal I/R. Mice (18–20 month, totally 5 mice) were deeply anesthetized and immobilized with a small stereotactic instrument, followed by exposure of their skulls and identification of their bregma. A 22 G needle was then used to drill a hole above the superior colliculus of each hemisphere, and 1 μl of 4% hydroxystilbamidine in PBS (aka Fluoro-Gold; Cat # ab138870, Abcam, USA) was injected into both superior colliculi, using a micro-injector, at 1 mm from the bony surface of the brain. Mice were then sacrificed at days 3 and 7 post-I/R, and retinal flat mounts were prepared. Fluoro-Gold-positive ganglion cells were identified under a fluorescent microscope, and quantitated by ImageJ.

### Luciferase reporter assay

To determine the possible target binding sequences of miR-150-5p on MEKK3 (MAP3K3), targetScan (http://www.targetscan.org/) was used. Both wild-type and mutant pMIR-MAP3K3-3’UTR luciferase reporter plasmids (Guangzhou RiboBio Co., Ltd, China) were obtained for the luciferase reporter assay, in which these plasmids were co-transfected with either miR-150-5p mimic, or negative control (NC) comprising of scrambled random miRNA, into 293T cells, using Lipofectamine 3000 (Thermo Fisher, USA). Dual-luciferase activity was then measured using the Dual-Glo Luciferase Assay System (Promega, USA).

### Statistical analysis

All statistical analyses was conducted using GraphPad Prism version 8.0, and all values were presented as mean ± standard error of the mean (SEM). Student’s t-test was used for comparisons between 2 groups, while for 3 or more groups, one way analysis of variance (ANOVA) was used. Additionally, comparisons to “baseline” measurements was defined in terms of changes pre- and post-I/R injury, while comparisons to “Normal” was in terms of differences between un-injured versus I/R-injured mice. p < 0.05 was considered statistically significant.

## Results

### Characterization of BM Sca-1^+^ and Sca-1^−^ exosomes

**Fig. 1:**
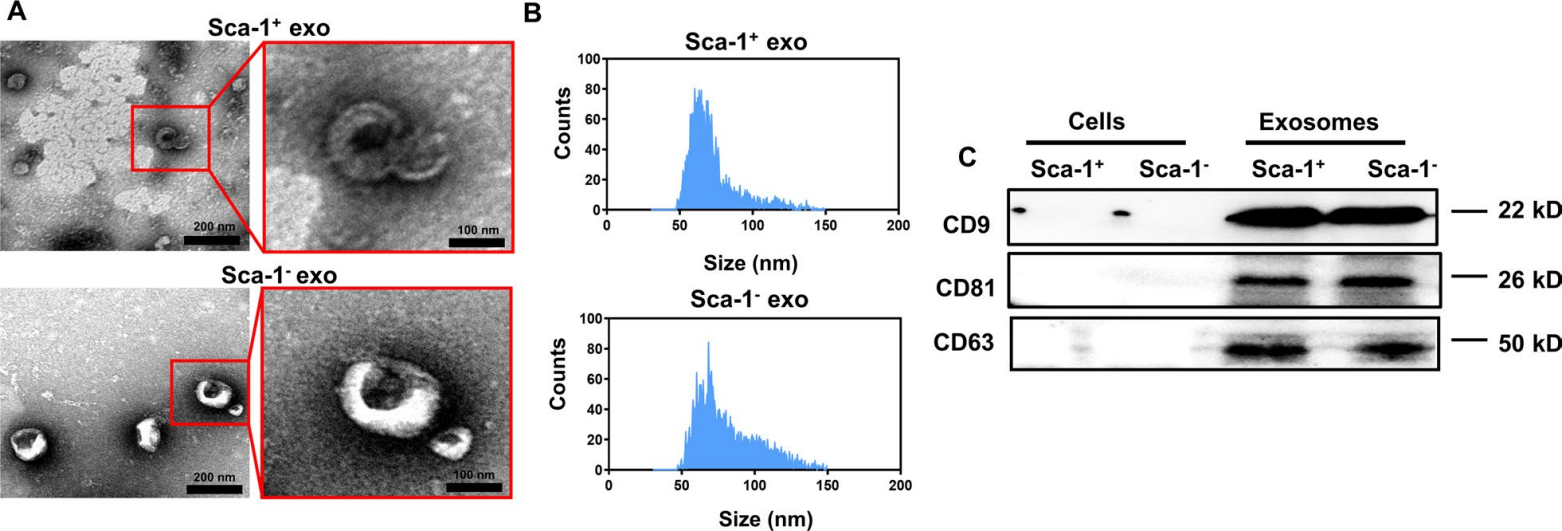
Characterization of bone marrow (BM) cell-derived exosomes. **A** Transmission electron microscopic (TEM) images of ring-shaped BMC-Sca-1^+^ and BMC-Sca-1^−^ exosomes. **B** Nanoparticle Tracking Analysis (NTA) histograms demonstrating the size distribution for BMC-Sca-1^+^ and BMC-Sca-1^−^ exosomes. **C** Western blot illustrating the characteristic surface markers CD63, CD9 and CD81 being present on BMC-Sca-1^+^ and BMC-Sca-1^−^ exosomes, unlike with cells

Western blot analyses confirmed successful isolation of BM Sca-1^+^ and Sca-1^−^ cells, as well as exosomes (Additional file 2: Fig. S1). Exosomes were then characterized using TEM, NTA and Western blot, where we found that exosomes from both cell types had typical round or cup-shaped morphologies, and were approximately 80 nm in diameter (Fig. 1A). A bell-shape distribution curve for exosome sizes were found for both cell types under NTA, indicating that most exosomes fell within the characteristic size range of 50–150 nm, with the highest peak at 70 nm (Fig. 1B). Western blot analysis showed that those exosomes expressed surface markers CD9, CD81, and CD63, all of which were absent in Sca-1^+^ or Sca-1^−^ cells (Fig. 1C). Therefore, these findings demonstrated successful isolation of exosomes from both Sca-1^+^ and Sca-1^−^ cells, adhering to the characteristic morphology, size, and surface marker profiles.

### BM Sca-1^+^ exosomes improved visual behavior in I/R induced retinal damage

**Fig. 2:**
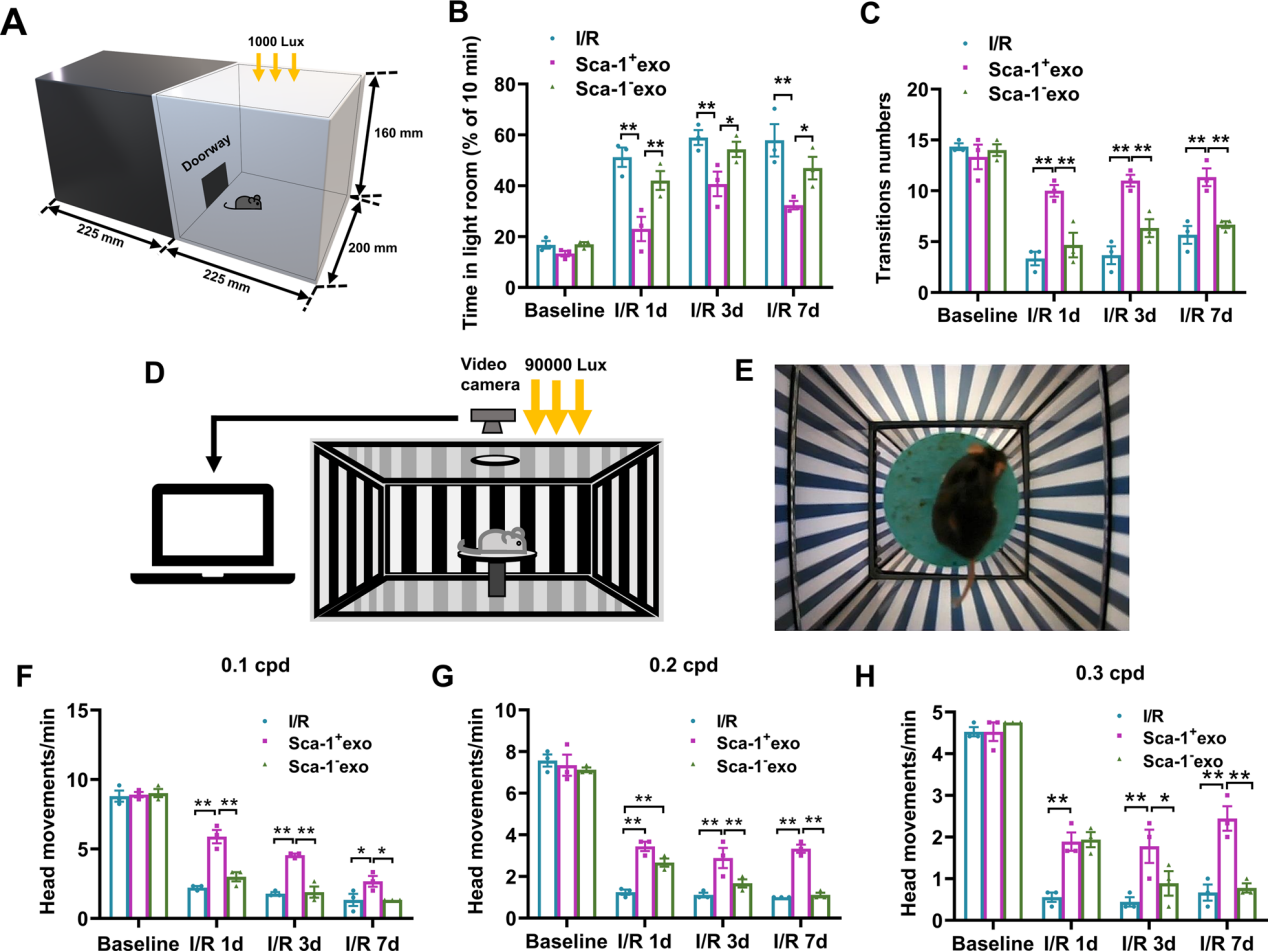
BM Sca-1^+^ exosomes improved visual behavior after retinal ischemia–reperfusion (I/R) injuries. **A** Schematic illustration of the apparatus for the light/dark exploration mouse model. **B, C** Mice who received Sca-1^+^ exosomes after I/R (I/R + Sca-1^+^ exo) were more able to respond to light exposure, compared to those who received Sca-1^−^ exosomes (I/R + Sca-1^−^ exo), or untreated post-I/R. Schematic illustration (**D**) and photograph (**E**) depicting the experimental apparatus for optomotor tests. **F–H** Visual behaviors, in terms of head movements, under 0.1–0.3 cycles per degree (cpd) were better preserved among I/R + Sca-1^+^ exo mice, compared to I/R and I/R + Sca-1^−^ exo. Data shown as mean ± SEM. n = 3 mice/group for all experiments. **P < 0.01, *P < 0.05

We first confirmed that exosomes from both Sca-1^+^ or Sca-1^−^ cells could be taken up by mouse retinal cells and found that they aggregated in the ganglion cell layer (Additional file 3: Fig. S2). The light/dark box exploration test was used to examine mouse visual behavior post-retinal I/R, with or without intravitreal injection of exosomes (Fig. 2A), where it was found that at baseline prior to I/R injury, all 3 animal groups (I/R, I/R + Sca-1^+^ exo, I/R + Sca-1^−^ exo) preferred to remain in the dark room, and had similar durations in the light room and transition numbers between dark and light rooms (Fig. 2B, C). At 1, 3, and 7 days post-I/R, though, all 3 groups spent longer durations in the light room, and had lower transition numbers (Fig. 2B, C). However, the I/R + Sca-1^+^ exo group had the shortest duration in the light room, and the highest transition numbers, compared to the other 2 groups during those time periods (Fig. 2B, C).

Another test for examining visual behavior was the optomotor response test, entailing the quantification of the number of head movements, under photopic conditions, during the rotation of the grating (Fig. 2D–E). The number of head movements/min, serving as a measure of visual function, decreased in all 3 groups following I/R compared to baseline, no matter the frequency of grating rotation (Fig. 2F–H). However, I/R + Sca-1^+^ exo had significantly higher head movements at days 3 and 7 days post-I/R, compared to the other 2 groups, at all 3 frequencies, though these differences were not present between I/R + Sca-1^+^ exo and I/R + Sca-1^−^ exo groups, at 0.2–0.3 cycles/degree (cpd), for day 1 post-I/R (Fig. 2F–H). All these findings thus demonstrate that the application of Sca-1^+^-derived exosomes resulted in greater maintenance of proper visual behaviors post-I/R, in terms of light/dark box exploration and optomotor responses.

### Sca-1^+^ exosomes protect retinal morphology by reducing I/R-induced ganglion cell apoptosis

**Fig. 3:**
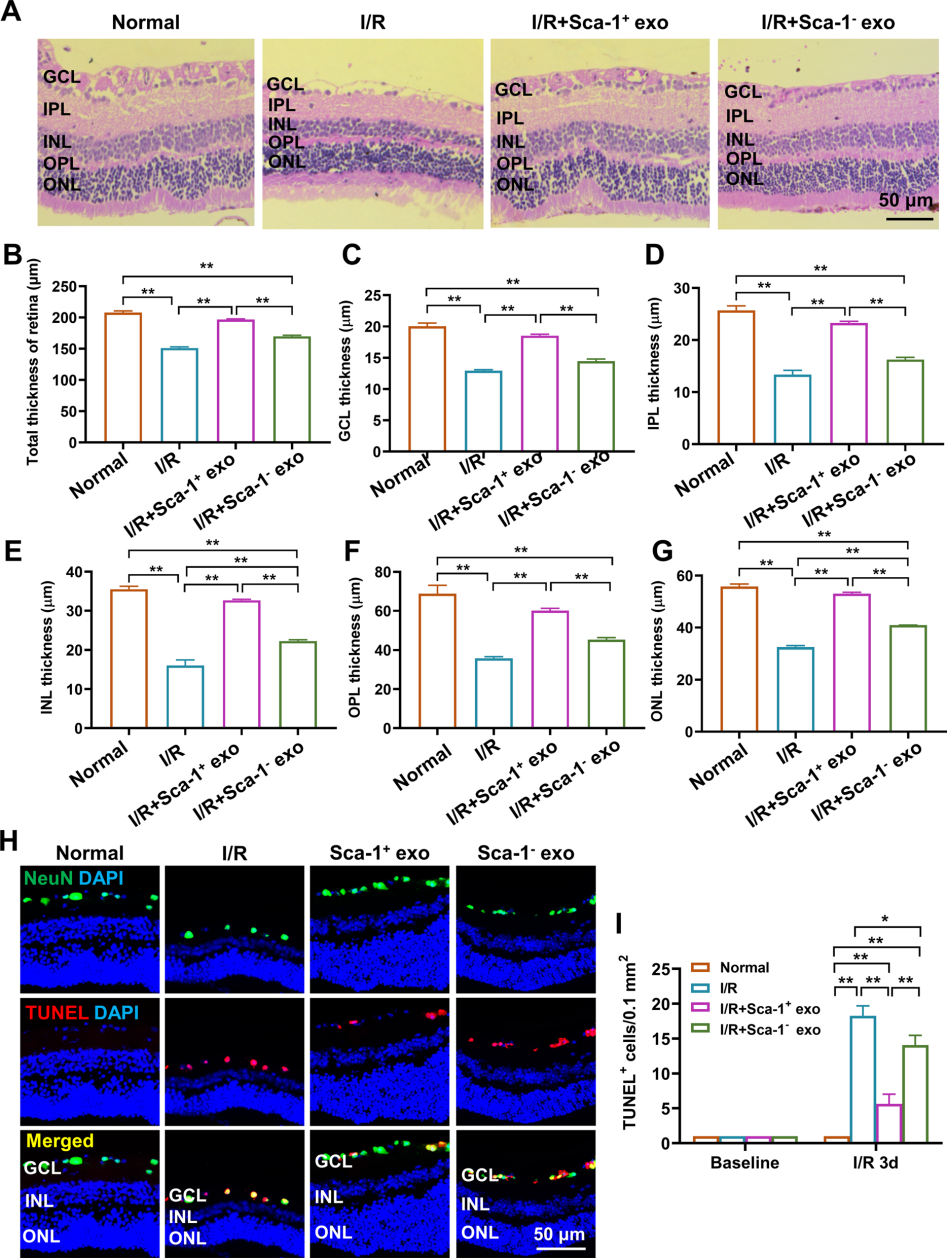
Sca-1^+^ exosomes protect retinal morphology by reducing I/R-induced retinal ganglion cell apoptosis. **A** Hematoxylin and eosin (H&E) staining of the retina to evaluate total **B**, ganglion cell (GCL; **C**), inner plexiform (IPL; **D**), inner nuclear (INL; **E**), outer plexiform (OPL; **F**), and outer nuclear (ONL; **G**) layer thicknesses, among control (Normal), I/R, I/R + Sca-1^+^ exo, and I/R + Sca-1^−^ exo groups. Representative immunofluorescence images (**H**) and quantification **I** of apoptotic (TUNEL^+^) retinal neurons (NeuN^+^), both at baseline and at 3 days post-I/R. Data shown as mean ± SEM. n = 3 retinas/group for all experiments. **P < 0.01, *P < 0.05

H&E staining was used to examine retinal morphology for uninjured normal (Normal), I/R, I/R + Sca-1^+^ exo, and I/R + Sca-1^−^ exo groups (Fig. 3A), where it was found that for both the entire retina, as well as the 5 retinal layers of ganglion cell (GCL), inner plexiform (IPL), inner nuclear (INL), outer plexiform (OPL) and outer nuclear layers (ONL), they were all thinner in I/R, compared to Normal (Fig. 3B–G). However, the administration of Sca-1^+^ exosomes in the I/R + Sca-1^+^ exo group yielded retinal thicknesses, both in total and for the 5 layers, closer to that of Normal, compared to I/R and I/R + Sca-1^−^ exo groups (Fig. 3B–G). To further examine the basis behind retinal layer thinning post-I/R, TUNEL was performed at 3 days post-I/R, where it was found that compared to baseline, significant increases in TUNEL^+^/NeuN^+^ cells were present among I/R, I/R + Sca-1^+^ exo, and I/R + Sca-1^−^ exo groups, compared to Normal (Fig. 3H, I). However, I/R + Sca-1^+^ exo had significantly fewer TUNEL^+^/NeuN^+^ cells, compared to the other 2 groups (Fig. 3H–I). In line with the findings of TUNEL^+^/NeuN^+^ cells being localized in GCL, Fluro-Gold labeling of viable retinal ganglion cells indicated that their numbers significantly decreased at days 3 and 7 post-I/R, compared to baseline, for I/R, I/R + Sca-1^+^ exo, and I/R + Sca-1^−^ exo groups. Notably, though, I/R + Sca-1^+^ exo had significantly higher numbers of Fluro-Gold^+^ cells, compared to the other 2 groups (Additional file 4: Fig. S3A, B). All of these findings thus indicate that intravitreal injection of Sca-1^+^ exosomes was able to preserve proper retinal layer morphology via lowering I/R-induced GCL cell apoptosis in aged mouse retinas.

### Sca-1^+^ exosomes reduced post-I/R M1 microglial polarization

**Fig. 4:**
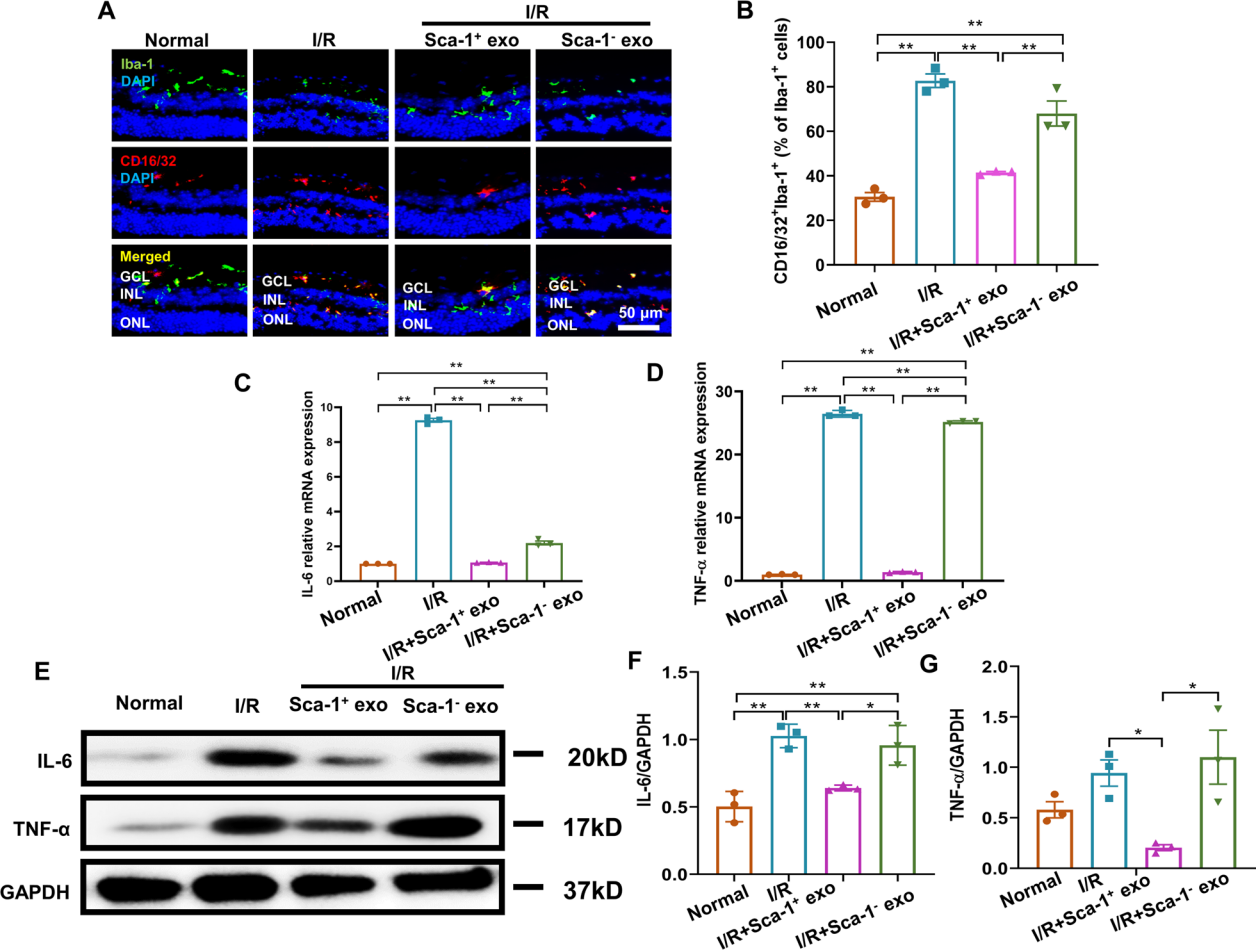
Sca-1^+^ exosomes reduced the occurrence of post-I/R microglial M1 polarization. Representative immunofluorescence images (**A**) and quantification (**B**) of M1 versus total microglia, excluding M1, among Normal, I/R, I/R + Sca-1^+^ exo, and I/R + Sca-1^−^ exo groups. Relative mRNA expression levels of inflammatory factors interleukin-6 (IL-6) (**C**) and tumor necrosis factor-α (TNF-α) (**D**) among the 4 treatment groups, as determined by reverse transcription-quantitative polymerase chain reaction (RT-qPCR). Representative Western blot image (**E**) and analysis of IL-6 (**F**) and TNF-α (**G**) protein expression levels among the 4 groups, normalized to glyceraldehyde 3-phosphate dehydrogenase (GAPDH). Data shown as mean ± SEM. n = 3 retinas/group for all experiments. **P < 0.01, *P < 0.05

I/R-induced retinal ganglion cell apoptosis may stem from M1 microglial polarization, as M1 microglia have previously been demonstrated to play important roles in post-I/R neurotoxicity [7]. Immunofluoresence staining of Iba-1, representing microglia, and CD16/32, representing M1 polarization, was thus performed, where it was found that compared to Normal, I/R, I/R + Sca-1^+^ exo, and I/R + Sca-1^−^ exo groups had higher percentages of M1 microglia (Fig. 4A, B). However, compared to the other 2 I/R groups, I/R + Sca-1^+^ exo had significantly less M1 polarization, being closer to that of Normal (Fig. 4A, B). The same trend was present among the 4 groups when examining retinal flatmounts, in which I/R, I/R + Sca-1^+^ exo, and I/R + Sca-1^−^ exo had higher M1 microglial percentages versus Normal; M1 microglial polarization levels among I/R + Sca-1^+^ exo was also lower than for I/R and I/R + Sca-1^−^ exo (Additional file 5: Fig. S4A, B).

As M1 polarization has also been associated with increased inflammation, we then examined the expression of pro-inflammatory cytokines IL-6 and TNF-α. We found higher levels of IL-6 and TNF-α, in terms of mRNA (Fig. 4C, D) and protein expression (Fig. 4E–G), in the I/R group. However, application of Sca-1^+^ exosomes lowered expression of these cytokines to levels closer to that of Normal, while Sca-1^−^ exosomes did not exhibit the same effect (Fig. 4C–G). Therefore, Sca-1^+^ exosome treatment reduced I/R-elicited M1 microglial polarization, which in turn led to lowered retinal mRNA and protein expression of pro-inflammatory IL-6 and TNF-α.

### miR-150-5p is enriched in Sca-1^+^ exosomes and regulates microglial polarization via repressing the MAP3K3 (MEKK3)/JNK signal pathway

**Fig. 5:**
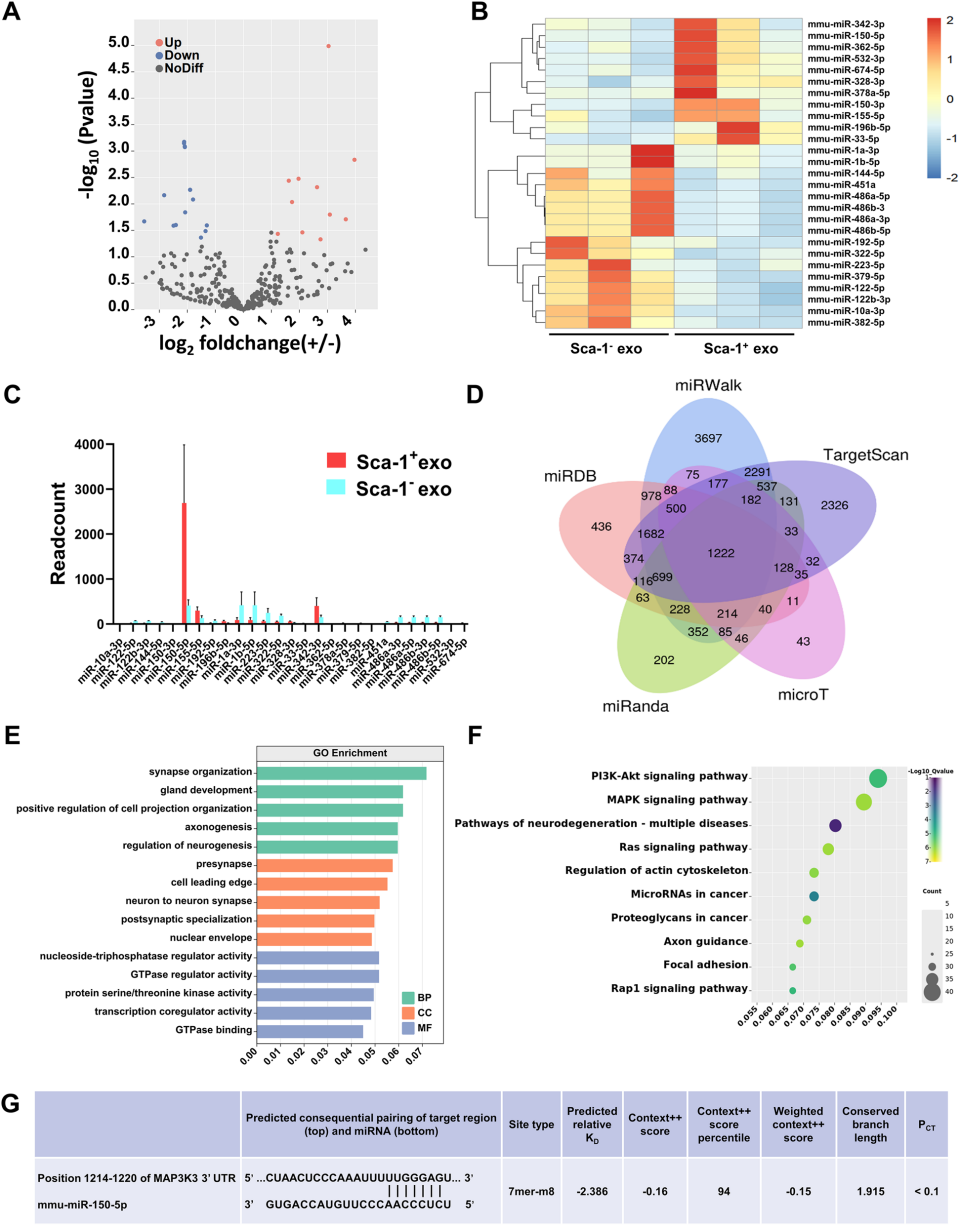
miR-150-5p is enriched within Sca-1^+^ exosomes and regulates microglial polarization via targeting MEKK3, the mouse analogue of mitogen-activated protein kinase kinase kinase 3 (MAP3K3). **A** Volcano plot showing miRNAs that are up- (red) and down (blue)-regulated in BM Sca-1^+^ exosomes, compared to Sca-1^−^ ones, as determined by high throughput sequencing. **B** Heatmap showing the 27 differentially-expressed miRNAs between the 2 groups, with up to twofold change in expression, of which 11 were up-regulated (warm colors, orange-red) and 16 down-regulated (cool colors, light–dark blue) in Sca-1^+^ versus Sca-1^−^ exosomes. **C** Relative abundance of the 27 miRNAs, in terms of read count, between the 2 groups. **D** Venn diagram demonstrating that the 27 differentially-expressed miRNA identified in our analysis were also those predicted by different databases. **E** Gene ontology (GO) enrichment analysis of the target genes for the 27 differentially-expressed miRNAs, in which they fell into 1 of 3 broad categories: biological process (BP), cellular component (CC), and molecular function (MF). **F** Kyoto genes and genomes (KEGG) enrichment analysis, in the form of a scatter plot, showing pathway associations for the target genes of these 27 miRNAs. **G** Predicted 3’-UTR binding site for miR-150-5p on the MEKK3 (MAP3K3) gene by TargetScan

Exosomes have been found to be enriched for miRNAs, which in turn are able to modulate the translation of target mRNAs [28]. To determine whether Sca-1^+^ exosomes were enriched in miRNAs, and if so, whether they are involved in modulating M1 microglial polarization, high-throughput sequencing was used to compare Sca-1^+^ and Sca-1^−^ exosome contents. As shown in the volcano plot (Fig. 5A), 486 miRNAs were detected, and significant differences in abundances between Sca-1^+^ and Sca-1^−^ exosome were found for 27 miRNAs. Out of those 27 miRNAs, 11 were significantly upregulated (fold change ≥ 1.0, p < 0.05), and 16 downregulated, in Sca-1^+^, compared to Sca-1^−^ exosomes (Fig. 5B). The most abundant miRNA in Sca-1^+^ exosomes was miR-150-5p, which was then examined in the following studies (Fig. 5C). To determine the downstream targets of the 27 differentially expressed miRNAs, 5 miRNA target prediction databases were used: miRDB, miRWalk, Targetscan, microT, and miRanda, based on the binding affinities of their 3′-UTR regions (Fig. 5D). The target genes were found under GO to be most enriched for synapse organization, pre-synapse, and nucleoside-triphosphatase regulator activity, with respect to biological process (BP), cellular component (CC), and molecular function (MF), respectively (Fig. 5E). KEGG analysis found that the top 10 most enriched signaling pathways were inflammation-related, such as PI3K-Akt, MAPK, and Ras. Additionally, neurodegeneration pathways were enriched, which was in accordance with I/R injury (Fig. 5F). With respect to miR-150-5p, we found that its target matched with the 3′-UTR of MAP3K3, the human version of MEKK3 in mice, which was part of the MAPK signaling pathway (Fig. 5G). These bioinformatics analyses thus suggest that the Sca-1^+^ exosomes may exert its effects, via miR-150-5p, to repress the MEKK3 signaling pathway, which may subsequently reduce M1 microglial polarization and inflammation.

### Increased miR-150-5p levels within Sca-1^+^ exosomes decreased M1 microglial polarization among BV2 cells in vitro via downregulating MEKK3/JNK signaling

**Fig. 6:**
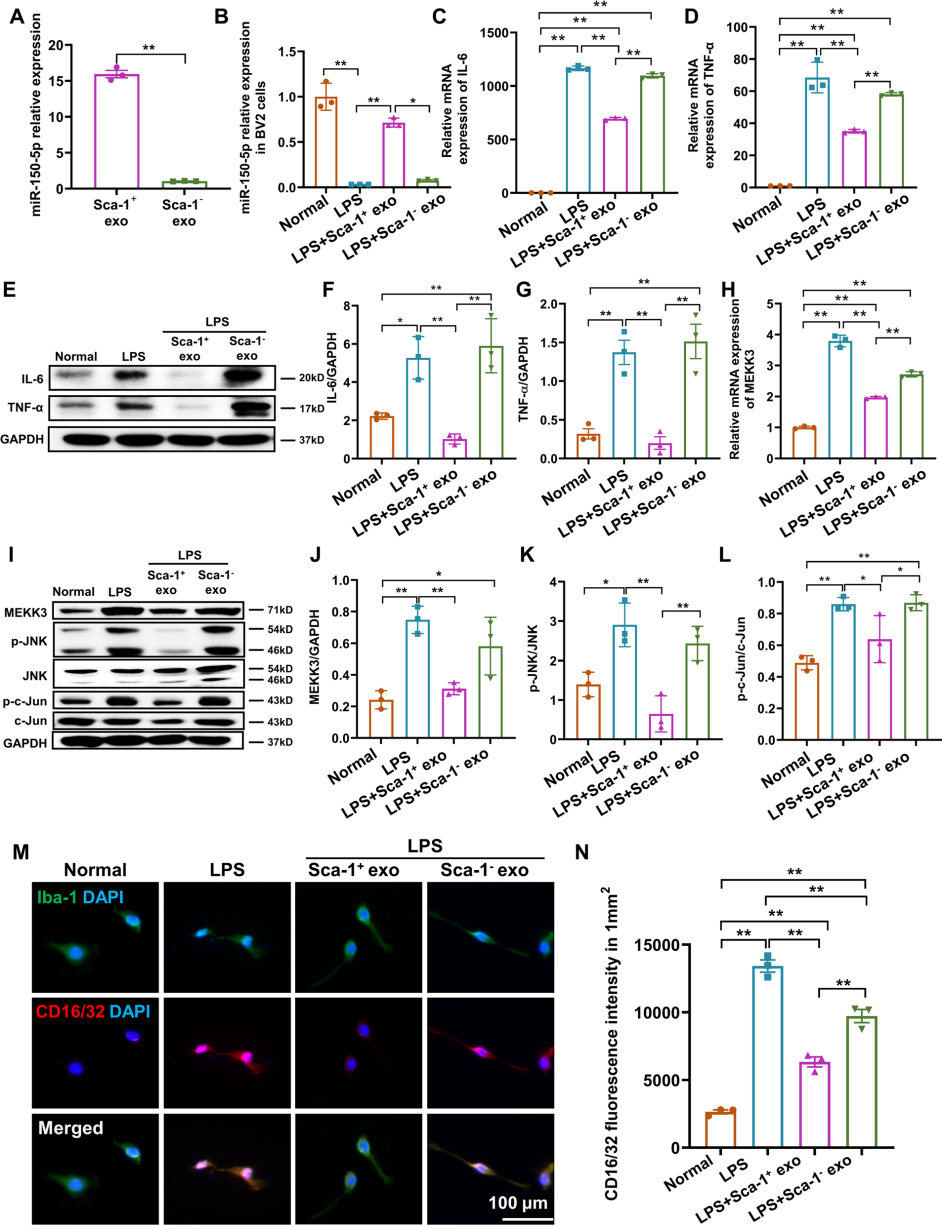
Increased miR-150-5p levels within Sca-1^+^ exosomes downregulate MEKK3/JNK signaling to decrease M1 microglial polarization among lipopolysaccharide (LPS)-induced BV2 microglial cells in vitro. **A** Relative expression levels for miR-150-5p within Sca-1^+^ and Sca-1^−^ exosomes, as determined by qPCR. **B** Relative expression levels of miR-150-5p within 4 BV2 cell treatment groups: Normal control, LPS, as well as Sca-1^+^ exosomes (LPS + Sca-1^+^ exo), or Sca-1^−^ exosomes (LPS + Sca-1^+^ exo) after LPS. Relative expression levels of inflammatory factors IL-6 (**C**) and TNF-α (**D**), as determined by RT-qPCR, among the 4 treatment groups. Representative Western blot images (**E**) and quantification of IL-6 (**F**) and TNF-α (**G**) protein expression levels among the 4 treatment groups, normalized to GAPDH. Relative expression levels of MEKK3 (**H**), the target gene of miR-150-5p, as determined by RT-qPCR, among the 4 treatment groups. Representative Western blot images (**I**) and quantification of MEKK3 (**J**), phosphorylated c-Jun N-terminal kinase (p-JNK)/JNK (**K**) and p–c-Jun/c-Jun (**L**) protein expression levels among the 4 treatment groups, normalized to GAPDH. Representative immunofluorescence images (**M**) and quantification (**N**) of mean CD16/32^+^ (M1 polarization biomarker) fluorescence intensity among BV2 cells in the 4 treatment groups. Data shown as mean ± SEM. n = 3/group for all experiments. **P < 0.01, *P < 0.05

To further validate whether Sca-1^+^ exosomes downregulated microglia M1 polarization via miR-150-5p, RT-qPCR was used to detect relative expression levels of miR-150-5p in Sca-1^+^ and Sca-1^−^ exosomes. There, we found that miR-150-5p expression was ~ 15 times higher in Sca-1^+^ versus Sca-1^−^ exosomes (Fig. 6A). Furthermore, we found that Sca-1^+^ and Sca-1^−^ exosomes could be taken up by BV2 cells, which was an immortalized murine microglial cell line, and no significant differences were found regarding the endocytosis of these 2 exosome types (Additional file 6: Fig. S5). Based on this finding, we established the LPS-induced inflammatory cell model, were we observed that LPS significantly reduced miR-150-5p expression in BV2 cells. This reduced expression, however, was able to be reversed back towards levels found in untreated (Normal) cells upon Sca-1^+^ exosome administration, but not for Sca-1^−^ exosomes (Fig. 6B). Furthermore, LPS significantly increased expression, both in terms of mRNA and protein levels, for IL-6 and TNF-α, compared to Normal. All of these levels, though, were significantly reduced in LPS + Sca-1^+^ exo, but not for LPS + Sca-1^−^ exo, whose levels were similar to that of LPS (Fig. 6C–G). These changes were most likely owed to miR-150-5p downregulating MEKK3, as LPS increased mRNA expression of MEKK3, which was lowered by Sca-1^+^, but not Sca-1^−^ exosome administration (Fig. 6H). Consistent with this finding, Western blot analyses showed that levels of MEKK3, and its downstream targets of p-JNK and p–c-Jun, significantly increased under LPS stimulation, compared to Normal. By contrast, all these proteins decreased in LPS + Sca-1^+^ exo, but not LPS + Sca-1^−^ exo group (Fig. 6I–L). To further confirm that MAP3K3 was the target gene of miR-150-5p, luciferase reporter assay was carried out, where it was found that luciferase activity decreased following co-transfection with miR-150-5p mimic and wild-type 3′-MAP3K3 UTR luciferase plasmid. By contrast, no changes in luciferase activity were present when the mimic was co-transfected with a luciferase plasmid containing mutant 3′-MAP3K3 UTR (Additional file 7: Fig. S6).

We then examined M1 polarization in BV2 cells using immunofluorescence staining for Iba-1 and CD16/32 markers, where the fluorescence intensity for CD16/32, representing M1 polarization among Iba-1^+^ BV2 cells, significantly increased in LPS versus Normal. However, applying Sca-1^+^ exosomes decreased CD16/32^+^ intensity to a greater extent in LPS + Sca-1^+^ exo, compared to LPS + Sca-1^−^ exo, indicating reduced M1 polarization (Fig. 6M, N). All of these findings from the LPS-stimulation model were in accordance with the results from retinal I/R, indicating that Sca-1^+^ exosomes could counteract against heightened inflammation, caused by I/R or LPS, via repressing the MEKK3/JNK/Jun pathway to decrease M1 microglial polarization.

### Increased miR-150-5p levels within Sca-1^+^ exosomes also decreased M1 microglial polarization in vivo via downregulating MEKK3/JNK signaling

**Fig. 7:**
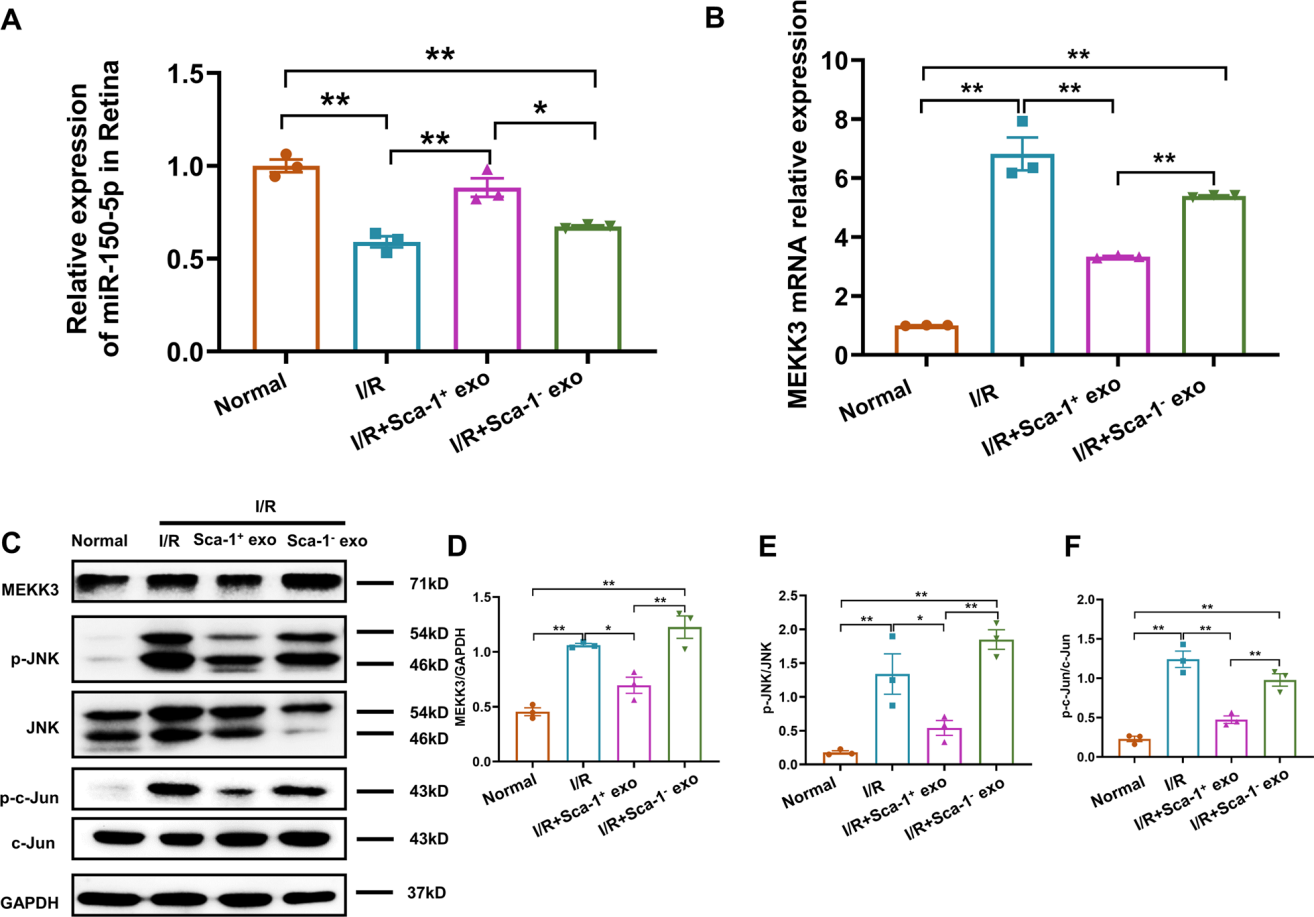
Increased miR-150-5p levels within Sca-1^+^ exosomes downregulate MEKK3/JNK signaling within post-I/R retinas in vivo. Relative expression of miR-150-5p (**A**) and MEKK3 (**B**) within the retina among the 4 treatment groups: Normal control, I/R, as well as Sca-1^+^ (I/R + Sca-1^+^ exo) or Sca-1^−^ (I/R + Sca-1^−^ exo) exosomes administered after I/R injury, as determined by RT-qPCR. Representative Western blot images (**C**) and quantification of MEKK3 (**D**), p-JNK/JNK (**E**), and p–c-Jun/c-Jun (**F**) protein expression levels among the 4 treatment groups, normalized to GAPDH. Data shown as mean ± SEM. n = 3/group for all experiments. **P < 0.01, *P < 0.05

Having shown in an in vitro model that Sca-1^+^ exosomes were able to downregulate the MEKK3/JNK/Jun pathway, and subsequent inflammatory factors, via miR-150-5p, we next aim to validate in vivo in old mouse retinas from an I/R injury model. We first examined miR-150-5p expression levels within the retinas of Normal, I/R, I/R, I/R + Sca-1^+^ exo, and I/R + Sca-1^−^ exo groups. As expected, I/R mice had significantly lower miR-150-5p levels, compared to Normal. However, I/R + Sca-1^+^ exo, restored miR-150-5p levels towards that of Normal, while miR-150-5p remained the same as I/R in the I/R + Sca-1^−^ exo group (Fig. 7A). MEKK3 expression, for both mRNA (Fig. 7B) and protein (Fig. 7C, D), exhibited the inverse pattern, in which I/R had significantly higher levels than that of Normal, and these levels significantly decreased upon Sca-1^+^, but not Sca-1^−^ exosome administration. The same pattern for MEKK3 was also found for the downstream targets p-JNK and p–c-JUN, where their protein levels were significantly higher in I/R, but decreased in IR + Sca-1^+^ exo. These decreases, however, did not occur in I/R + Sca-1^−^ exo group (Fig. 7E, F). The correspondence between in vitro and in vivo findings thus demonstrates that Sca-1^+^ exosomes may exert its post-injury anti-inflammatory effects, via miR-150-5p repressing the MEKK3/JNK/Jun pathway.

## Discussion

BM Sca-1^+^ stem cells from young donors have been noted to be able to aid in recovery and improve tissue functioning in aged recipients in multiple previous studies, such as one where old mice reconstituted with young Sca-1^+^ BM cells exhibited enhanced autophagy in aged hearts [29], as well as attenuated stroke-induced neurological dysfunction [30] and radiotherapy-induced cognitive impairments [31]. However, the usage of Sca-1^+^ stem cells is not without risk, particularly with respect to immune rejection and possible tumorigenesis stemming from radiation exposure prior to reconstitution [32]. Exosomes derived from those cells, though, could serve as a possible approach to mitigate these shortcomings, as they likely serve as the basis behind the beneficial paracrine support of Sca-1^+^. Indeed, over the past few years, exosome-based therapies have become a promising approach as a cell-free therapy for tissue repair [33]. In this study, we found that exosomes derived from young BM Sca-1^+^ cells were able to alleviate the effects of retinal I/R injury via reducing ganglion cell apoptosis. In particular, these exosomes were enriched for miR-150-5p, which repressed M1 microglial polarization and inflammatory responses, via suppressing MEKK3/JNK/Jun signaling. All of these changes, in turn, resulted in preserved visual functioning.

Previously, we had demonstrated that old chimeric mice containing young BM Sca-1^+^ stem cell had greater reparative capabilities post-retinal I/R [9], owing to the homing and differentiation of BM Sca-1^+^ cells into microglia in the retina. Furthermore, these cells had excellent neurotrophic capabilities, displaying high expression levels for various neurotrophic factors [34]. Based on these findings, as well as from multiple other studies showing that stem cell-derived exosomes could serve as a cell-free therapeutic alternative to traditional stem cell therapies [35], we hypothesized that young BM Sca-1^+^-derived exosomes may exert the same pro-reparative effects as BM Sca-1^+^ stem cell in the aged retina, and that these effects may also be associated with microglia polarization alterations. Size-exclusion chromatography was used to harvest secreted exosomes from cell culture. We found, consistent with a previous report, that this method was able to isolate exosomes at a high purity, along with being reproducible and scalable for large quantities [36].

Indeed, over the past few years, exosome-based therapies have become a promising approach as a cell-free therapy for tissue repair [33]. They have particularly shown promise when delivered into the retina via intravitreal injection. For instance, one study found that intravitreal injection of exosomes containing the anti-angiogenic peptide KV11 was more effective, compared to injecting KV11 alone, in suppressing pathological angiogenesis within the retina [17]. Furthermore, intravitreal injection of exosomes derived from mesenchymal stem cells were able to promote recovery of retinal laser injury, due to these exosomes down-regulating monocyte chemotactic protein (MCP)-1 expression [37]. In line with these findings, we found in this study that exosomes derived from young BM Sca-1^+^ cells were able to alleviate the effects of retinal I/R injury via reducing ganglion cell apoptosis.

To verify the therapeutic effect of Sca-1^+^ exosomes, the most intuitive approach is by using visual functional tests. One such test, the light/dark test, is based on the unconditioned preference of rodents for dark over bright environments. Thus, increases in their duration of stay in the light chamber is suggestive of diminished visual acuity [38]. We observed that I/R mice who received Sca-1^+^ exosomes showed robust aversion to light and greater movements, compared to untreated I/R mice, and I/R mice treated with Sca-1^−^ exosomes. These findings were also consistent with those from the optomotor response test, another simple and rapid method for assessing visual defects in mice [39]. Its operation is based on the fact that most animals turn their heads to stabilize the images on the retina, as a compensation method in a globally moving environment. A greater optomotor response, in the form of more involuntary head movements, was recorded in I/R mice who received Sca-1^+^ exosomes, indicating that they had better visual acuity.

On top of reduced visual functioning, retinal I/R was also found to be associated with decreased retinal thickness and GCL cell density, along with increased apoptosis [40]. In our study, these outcomes were reflected with increased TUNEL^+^, and decreased Fluoro-Gold^+^ cells, which represented, respectively, apoptotic [41] and live ganglion cells. All of these I/R-associated changes, however, were reversed upon Sca-1^+^ exosome administration, indicating that it exerted its reparative effects by reducing ganglion cell apoptosis, in turn preserving retinal thickness and structure. We also found that after intravitreal injection, exosomes aggregated in the ganglion. This aggregation pattern is likely due to the ganglion cell layer being the first cell layer exposed to intravitreal injection, which may trigger active endocytosis by this cell layer. It has been noted by Pollalis et al., though, that intravitreally-injected exosomes are able to enter other retinal layers, including inner plexiform, inner nuclear, outer plexiform, and outer nuclear layers [42]. However, exosome delivery in those layers was observed in the context of targeting choroidal neovascularization, which occurs below the retina; indeed, Pollalis et al. designed these exosomes to specifically target that region. This stands in contrast with our study, which focused on counteracting against I/R induced neuronal cell death within the retina itself.

I/R injury is also associated with neuroinflammation and pyroptosis, of which its mechanistic basis may be due to increased M1 microglia, which has been found post-injury in various organs, such as the brain [43, 44], spine [45], heart [46], and retina [47]; these M1 microglia, in turn, release pro-inflammatory cytokines. It has been previously documented, though, that one possible approach to attenuate neuroinflammation was by reducing M1 microglial activity, leading to lowered inflammatory factor production. This modulation of M1 microglia, in turn could be carried out via exosome exposure [48, 49]. Indeed, in our study, we found increased proportions of CD16/32^+^ cells, which represents pro-inflammatory M1 microglia [50], post-I/R injury. This increase in M1-type microglia was concomitant with elevated levels of pro-inflammatory cytokines IL-6 and TNF-α, both of which were likely secreted by activated M1 microglia in the retina. Furthermore, it was observed that enrichment of the inner retina with Sca-1^+^ exosomes may be associated with decreases in retinal M1 microglia in retina and inflammatory responses, suggesting that these exosomes may serve as a possible anti-inflammatory treatment when applied intravitreally.

To elucidate the underlying molecular mechanisms behind the reparative effects of Sca-1^+^ exosomes, bioinformatics and molecular analyses were performed. It has been documented in previous studies that miRNA could serve as a mediator responsible for the therapeutic effects of exosomes [51]. Our application of high-throughput sequencing technology helped us identify the putative exosomal miRNAs responsible for the pro-reparative phenotypes that we observed. We found that several hundred miRNAs were present within BM stem cell exosomes, and out of those differentially-expressed miRNAs, miR-150-5p emerged as a candidate of interest. miR-150-5p was previously associated with neurodegeneration, and was able to modulate matrix metalloproteinase-14 and vascular endothelial growth factor expression, serving as a possible therapeutic strategy for rheumatoid arthritis [52]. Furthermore, miR-150-5p in BM stem cell-derived exosomes was able to attenuate myocardial infarction in mice, via its targeting of Bcl-2-associated X protein [53], and slowed myocardial fibrosis progression by targeting early growth response 1 [54]. Additionally, it protected against septic acute kidney injury via downregulating MEKK3 [55]. In line with these findings, we found that miR-150-5p was most highly expressed in Sca-1^+^ versus Sca-1^−^ exosomes, and that its target genes were related to synapse organization, pre-synapse and neurogenesis under GO. As for signaling pathways, miR-150-5p was most strongly associated, under KEGG, with inflammation-related signaling pathways, and the strongest candidate pathway was MAPK signaling, due to MEKK3 being identified as a common target gene for miR-150-5p in 5 separate databases.

We then examined whether exosomes could deliver miRNAs into specific cells and tissues. As previously demonstrated, circulating monocytes could take up exosomes [56], and miRNAs within exosomes were able to be transferred to other types of cells [57]. This was supported by our findings, in which BV2 cells displayed bright red fluorescence, originating from Dil-labelled exosomal membranes [58], thereby indicating that these BV2 cells took up exosomes via endocytosis [59]. Microglial uptake of exosomes was further confirmed by increased abundance of miR-150-5p in BV2 cells after LPS exposure and Sca-1^+^ exosome administration, as miR-150-5p is usually barely expressed in LPS-stimulated BV2 cells. All these observations therefore demonstrated that Sca-1^+^ exosomes contained miRNA, particularly miR-150-5p, which could be transferred directly into target cells. The transfer of miRNA into microglia was further confirmed by the presence of significant down regulation of its downstream target gene, MEKK3, which was part of the MEKK3/JNK/c-Jun signaling pathway. MEKK3 downregulation, in turn, was associated with inhibition of JNK and c-Jun phosphorylation, ultimately leading to reduced expression of downstream inflammatory factors IL-6 and TNF-α. The repression of the MEKK3/JNK/c-Jun signaling pathway by miR-150-5p was further confirmed by the lack of CD16/32^+^, indicating M1 polarization, among LPS-activated BV2 cells co-cultured with in Sca-1^+^ exosomes. LPS exposure has been documented to polarize microglia towards an M1 phenotype [48], indicating that Sca-1^+^ exosomes inhibited this polarization in BV2 cells, via delivering miR-150-5p, which subsequently downregulated the MEKK3/JNK/c-Jun signal pathway. This in vitro finding was verified in vivo in a I/R mouse model, in which I/R mice who received Sca-1^+^ exosomes had increased miR-150-5p levels, as well as lowered MEKK3, p-JNK, and p–c-JUN. It should be noted, though, that miR-150-5p may not be the only factor behind the neuroprotective effects of Sca-1^+^ exosomes. Indeed, we found that 27 miRNAs had significant differences in expression levels between Sca-1^+^ and Sca-1^−^ exosomes, and that 11 of those were significantly up-regulated in Sca-1^+^ exosomes. Therefore, even though miR-150-5p was the most up-regulated in Sca-1^+^ exosomes, the other 10 miRNAs could also contribute to the neuroprotective effect. For instance, miR-21 has been documented by Deng et al. to exert protective effects against retinal degeneration, particularly via counteracting against photoreceptor apoptosis [60]. Therefore, future studies should be conducted to determine the potential neuroprotective roles that these other exosomal miRNAs may play.

## Conclusion

In this study, we found that exosomes produced by young BM Sca-1^+^ stem cells were able to counteract against the effects of retinal I/R injury, due to them being enriched for miR-150-5p, compared to those obtained from Sca-1^−^ cells. miR-150-5p acted upon retinal microglia to repress the MEKK3/JNK/c-Jun pathway, resulting in reduced M1 microglial polarization, and subsequent lowered expression of pro-inflammatory cytokines IL-6 and TNF-α, all of which ultimately yielded decreased ganglion cell apoptosis, as well as preservation of proper retinal morphology and visual functioning. Therefore, Sca-1^+^ exosomes could serve as a possible cell-free treatment approach for retinal I/R injury.

## Supplementary Information

**Additional file 1: Table S1**. Primer sequences used in the study.

**Additional file 2: Fig. S1:** Western blot illustrating the specificity of stem cell antigen-1 (Sca-1) expression on magnetic bead-sorted Sca-1^+^ cells and exosomes, which was absent from Sca-1^−^ cells and exosomes.

**Additional file 3: Fig. S2:** Representative immunofluorescence images of bone marrow stem cell-derived Sca-1^+^ and Sca-1^−^ exosomes (Sca-1^+^ and Sca-1^−^ exo), labelled with Dil dye (red) within the ganglion cell layer (GCL) of the mouse retina, labelled with NeuN (green), at 0, 12, 24, and 48-h post-exosome injection. INL: inner nuclear layer, ONL: outer nuclear layer.

**Additional file 4: Fig. S3:** Representative images (**A**) and quantification (**B**) of viable retinal ganglion cells, labelled by Fluoro-Gold, in I/R, as well as bone marrow stem cell-derived Sca-1^+^ and Sca-1^−^ exosome groups (IR + Sca-1^+^ and I/R + Sca-1^−^ exo), at baseline, 3 and 7 days after I/R injury. Data shown as mean ± standard error of the mean (SEM). n = 3/group. **P < 0.01, *P < 0.05.

**Additional file 5: Fig. S4:** Sca-1^+^ exosomes reduced the occurrence of post-I/R microglial M1 polarization. Representative immunofluorescence images in retinal flatmounts (**A**) and quantification (**B**) of M1 versus total microglia, excluding M1, among Normal, I/R, I/R + Sca-1^+^ exo, and I/R + Sca-1^−^ exo groups. Data shown as mean ± SEM. n = 3/group. **P < 0.01.

**Additional file 6: Fig. S5:** Endocytosis of Sca-1^+^ and Sca-1^−^ exosomes (represented by Dil dye, red) by BV2 cells (Iba-1^+^, green), compared to Control without exosome treatment.

**Additional file 7: Fig. S6:** Mitogen-activated protein kinase kinase kinase 3 (MAP3K3) was a direct target of miR-150-5p. **A** Schematic diagram showing miR-150-5p base-pairing with wild-type (WT), but not with mutant (MUT) versions of the 3’ UTR binding site of MAP3K3. **B** Luciferase activity decreased following co-transfection with miR-150-5p mimic and wild-type 3’-MAP3K3 UTR luciferase plasmid, while no changes were present following co-transfection of the mimic with mutant luciferase plasmid. Luciferase activity in miR-150-5p negative control (NC, comprising scrambled control miRNA) was set at 1.0. Data shown as mean ± SEM. n = 3/group, **p < 0.01.

## Data Availability

The datasets used and analyzed during our study are available from the first authors on reasonable request.

## Abbreviations

ANOVA: Analysis of variance
BM: Bone marrow
BP: Biological process
CC: Cellular component
CD: Cluster of differentiation
cpd: Cycles/degree
DAPI: 4′,6-Diamidino-2-phenylindole
FITC: Fluorescein
GCL: Ganglion cell layer
GO: Gene Ontology
H&E: Hematoxylin and eosin
Iba-1: Ionized calcium-binding adapter molecule 1
I/R: Ischemia–reperfusion injury
IgG: Immunoglobulin G
IL: Interleukin
INL: Inner nuclear layer
IOP: Intraocular pressure
IPL: Inner plexiform layer
KEGG: Kyoto Encyclopedia of Genes and Genomes
LPS: Lipopolysaccharide
MEKK3: Mitogen-activated protein kinase kinase kinase 3
MF: Molecular function
ncRNA: Non-coding ribonucleic acid
NTA: Nanoparticle tracking analysis
ONL: Outer nuclear layer
OPL: Outer plexiform layer
PBS: Phosphate buffered saline
PFA: Paraformaldehyde
Sca-1: Stem cell antigen 1
SEM: Standard error of the mean
TEM: Transmission electron microscopy
TNF: Tumor necrosis factor
TUNEL: Terminal deoxynucleotidyl transferase dUTP nick end labeling assay
