# Correction to: Young Sca-1 + bone marrow stem cell-derived exosomes preserve visual function via the miR-150-5p/MEKK3/JNK/c-Jun pathway to reduce M1 microglial polarization

**DOI:** 10.1186/s12951-023-01964-6

**Published:** 2023-07-12

**Authors:** Yuan Wang, Wan-yun Qin, Qi Wang, Xin-na Liu, Xiang-hui Li, Xin-qi Ye, Ying Bai, Yan Zhang, Pan Liu, Xin-lin Wang, Yu-hang Zhou, Hui-ping Yuan, Zheng-bo Shao

**Affiliations:** 1 Department of Ophthalmology, The Second Affiliated Hospital of Harbin Medical University, Harbin, China; 2 Future Medical Laboratory, the Second Affiliated Hospital of Harbin Medical University, Harbin, China; 3 The Key Laboratory of Myocardial Ischemia, Harbin Medical University, Ministry Education, Harbin, China

**Journal of Nanobiotechnology 2023 21:194**

10.1186/s12951-023-01944-w

Following publication of the original article [1], the order that the authors appeared in the author list was incorrect.

The author group has been updated above and the original article [1] has been corrected.
