# Supplementary material for: Young Sca-1+ bone marrow stem cell-derived exosomes preserve visual function via the miR-150-5p/MEKK3/JNK/c-Jun pathway to reduce M1 microglial polarization
Source: J Nanobiotechnology. 2023 Jun 15;21:194. doi: 10.1186/s12951-023-01944-w (PMC10268362; doi:10.1186/s12951-023-01944-w)
Supplement: Supplementary file 1 — Additional file 1: Table S1. Primer sequences used in the study. [file 12951_2023_1944_MOESM1_ESM.docx]

**Table S1**. Primer sequences used in the study.

| Gene | Forward | Reverse |
| --- | --- | --- |
| IL-6 | 5’-TAGTCCTTCCTACCCCAATTTCC-3’ | 5’-TTGGTCCTTAGCCACTCCTTC-3’ |
| TNF-α | 5’-CCCTCACACTCAGATCATCTT CT-3’ | 5’-GCTACGACGTGGGCTACAG-3’ |
| MEKK3 | 5’-CTTGGGCAGAGTATGAAGCTAT-3’ | 5’-CTGACGAGCTTCCACAAATATG-3’ |
| GAPDH | 5’-CCAAGGAGTAAGACCCCTGG-3’ | 5’-TGGTTGAGCACAGGGTACTT-3’ |
| miR-150-5p | 5’-GCGTCTCCCAACCCTTGTACCAGTG-3’ |  |
| U6 | 5’-AGAGAAGATTAGCATGGCCCCTG-3’ |  |
