# Supplementary figures and images for: Young Sca-1+ bone marrow stem cell-derived exosomes preserve visual function via the miR-150-5p/MEKK3/JNK/c-Jun pathway to reduce M1 microglial polarization
Source: J Nanobiotechnology. 2023 Jun 15;21:194. doi: 10.1186/s12951-023-01944-w (PMC10268362; doi:10.1186/s12951-023-01944-w)

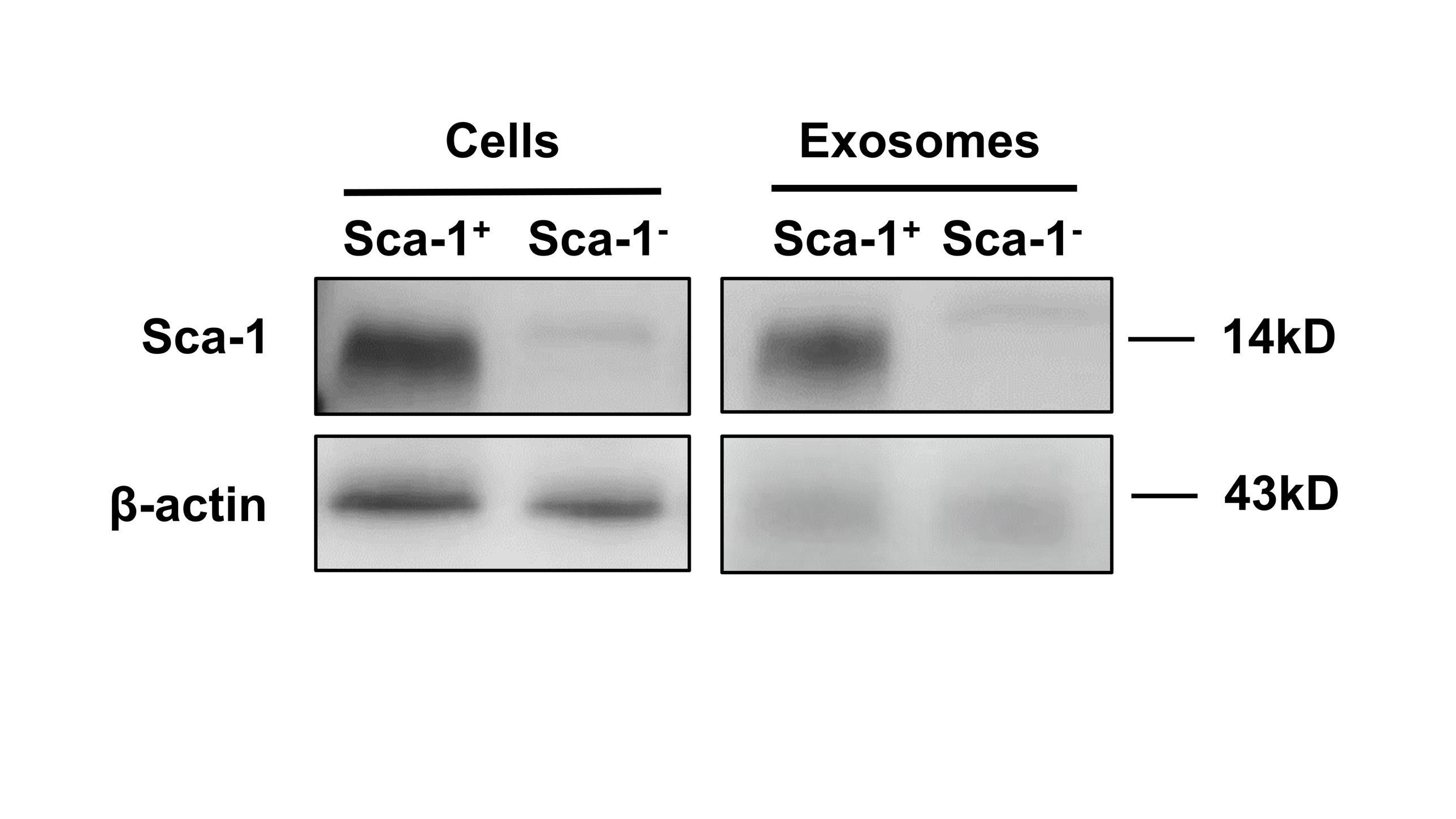

Supplement: Supplementary file 2 — Additional file 2: Fig. S1: Western blot illustrating the specificity of stem cell antigen-1 (Sca-1) expression on magnetic bead-sorted Sca-1+ cells and exosomes, which was absent from Sca-1− cells and exosomes. [file 12951_2023_1944_MOESM2_ESM.tif]

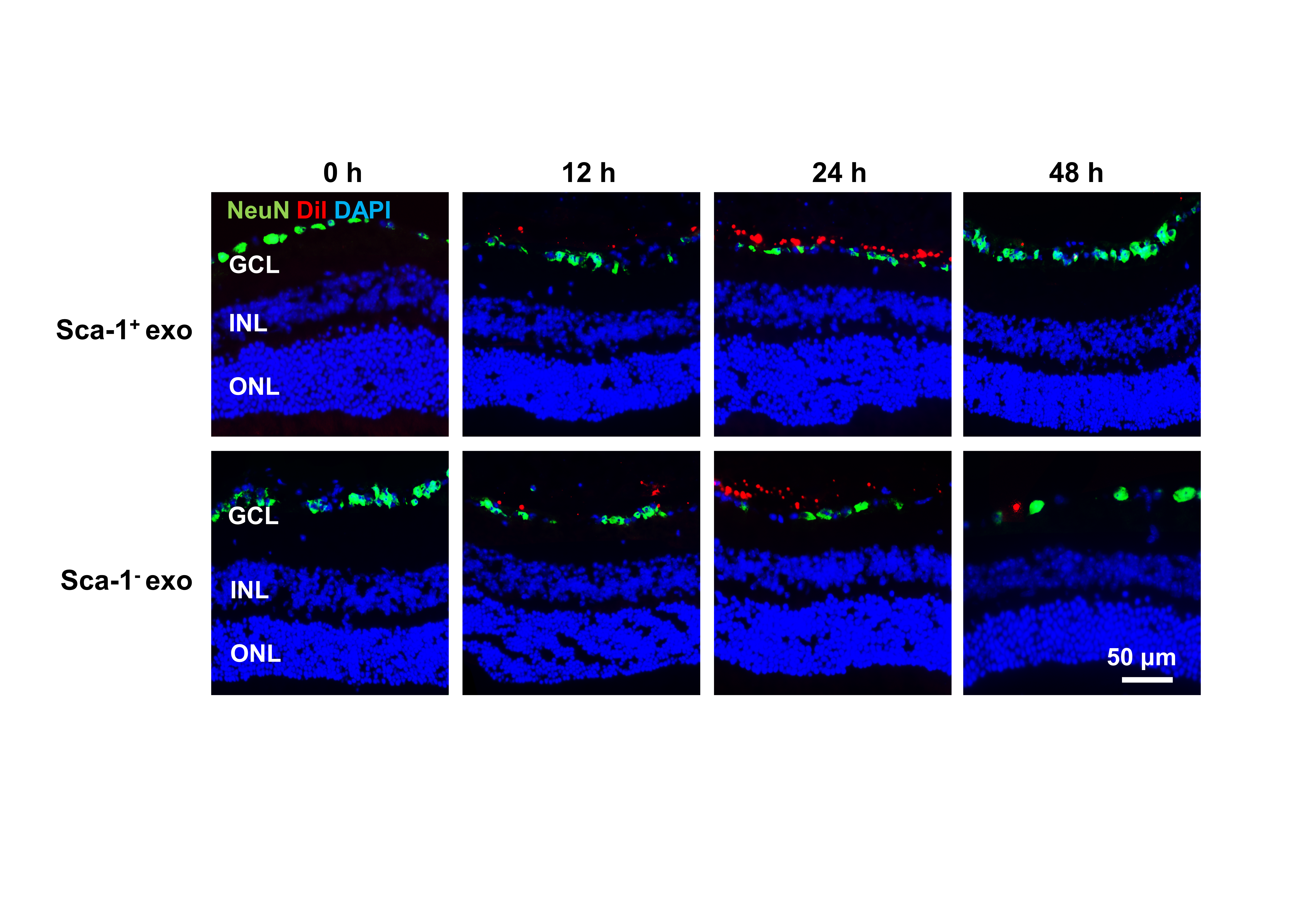

Supplement: Supplementary file 3 — Additional file 3: Fig. S2: Representative immunofluorescence images of bone marrow stem cell-derived Sca-1+ and Sca-1− exosomes (Sca-1+ and Sca-1− exo), labelled with Dil dye (red) within the ganglion cell layer (GCL) of the mouse retina, labelled with NeuN (green), at 0, 12, 24, and 48-h post-exosome injection. INL: inner nuclear layer, ONL: outer nuclear layer. [file 12951_2023_1944_MOESM3_ESM.tif]

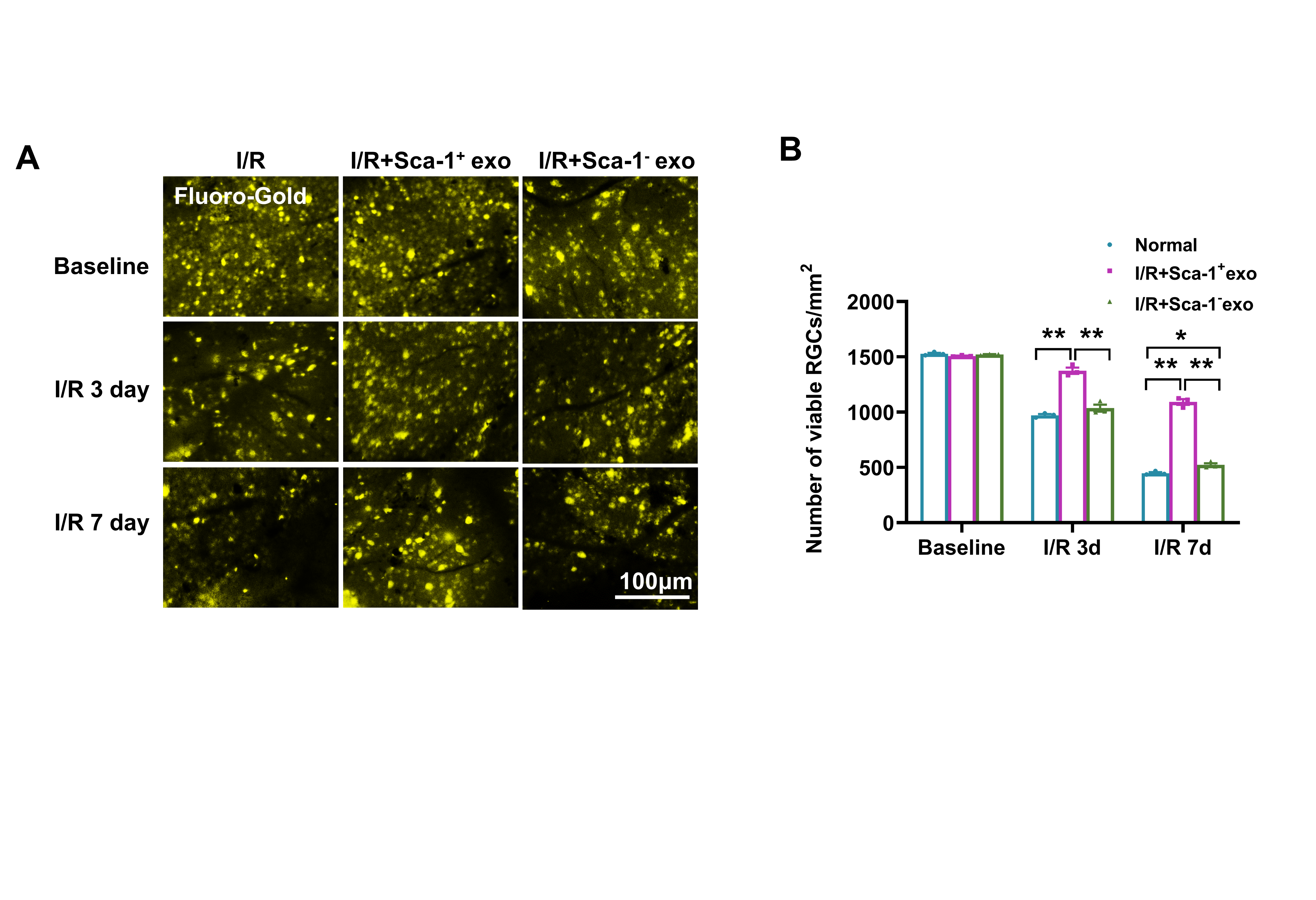

Supplement: Supplementary file 4 — Additional file 4: Fig. S3: Representative images (A) and quantification (B) of viable retinal ganglion cells, labelled by Fluoro-Gold, in I/R, as well as bone marrow stem cell-derived Sca-1+ and Sca-1− exosome groups (IR + Sca-1+ and I/R + Sca-1− exo), at baseline, 3 and 7 days after I/R injury. Data shown as mean ± standard error of the mean (SEM). n = 3/group. **P < 0.01, *P < 0.05. [file 12951_2023_1944_MOESM4_ESM.tif]

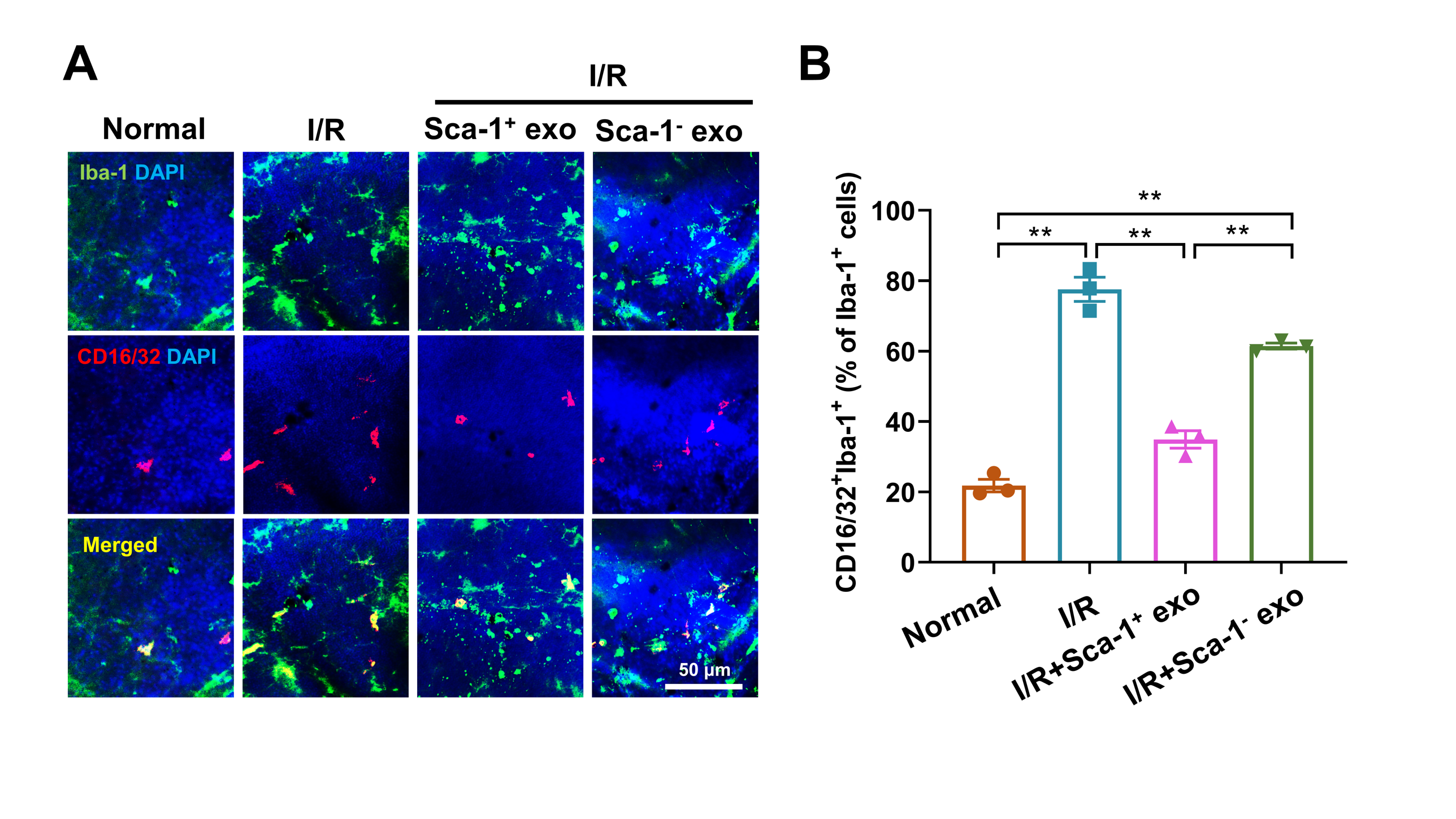

Supplement: Supplementary file 5 — Additional file 5: Fig. S4: Sca-1+ exosomes reduced the occurrence of post-I/R microglial M1 polarization. Representative immunofluorescence images in retinal flatmounts (A) and quantification (B) of M1 versus total microglia, excluding M1, among Normal, I/R, I/R + Sca-1+ exo, and I/R + Sca-1− exo groups. Data shown as mean ± SEM. n = 3/group. **P < 0.01. [file 12951_2023_1944_MOESM5_ESM.tif]

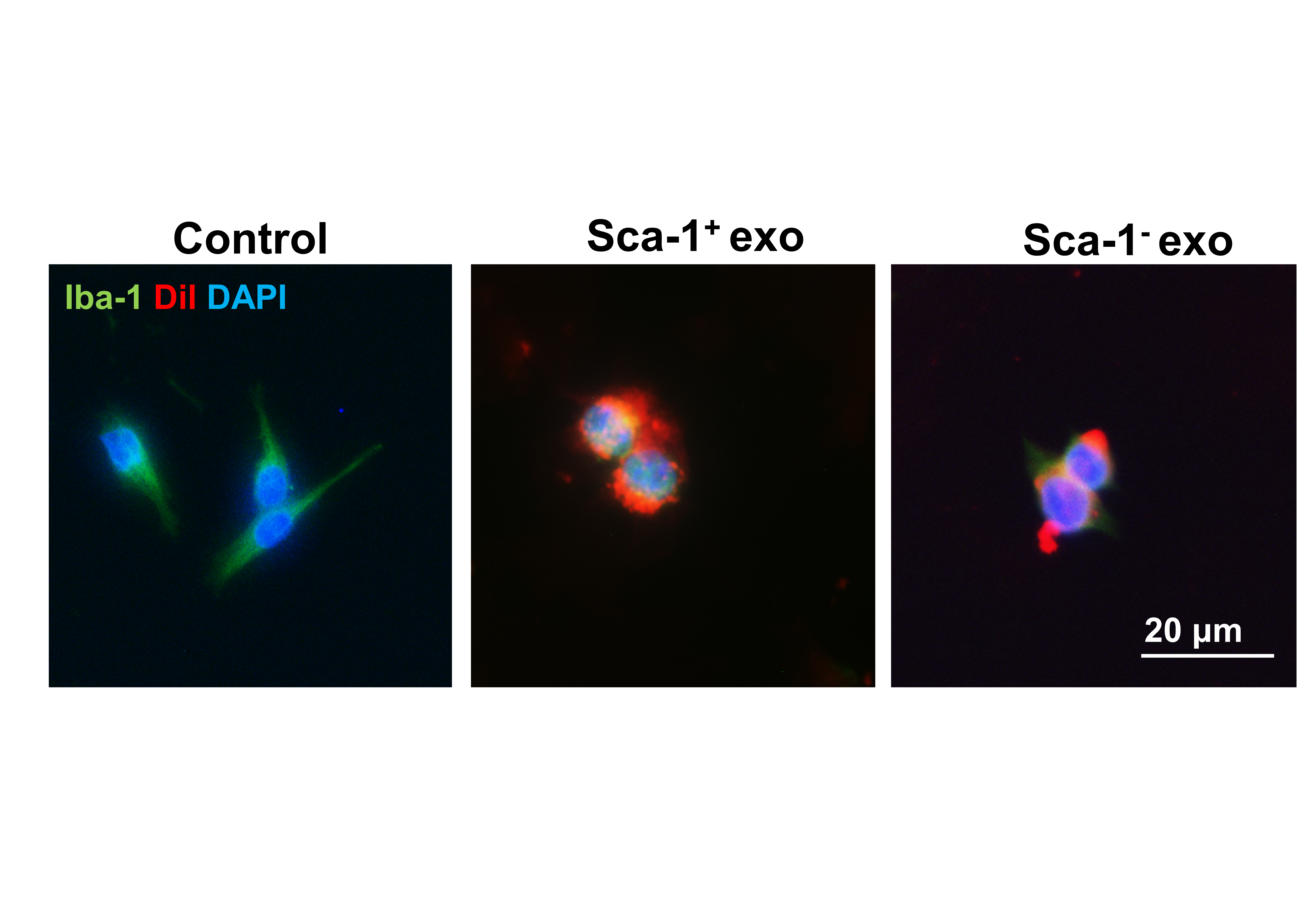

Supplement: Supplementary file 6 — Additional file 6: Fig. S5: Endocytosis of Sca-1+ and Sca-1− exosomes (represented by Dil dye, red) by BV2 cells (Iba-1+, green), compared to Control without exosome treatment. [file 12951_2023_1944_MOESM6_ESM.tif]

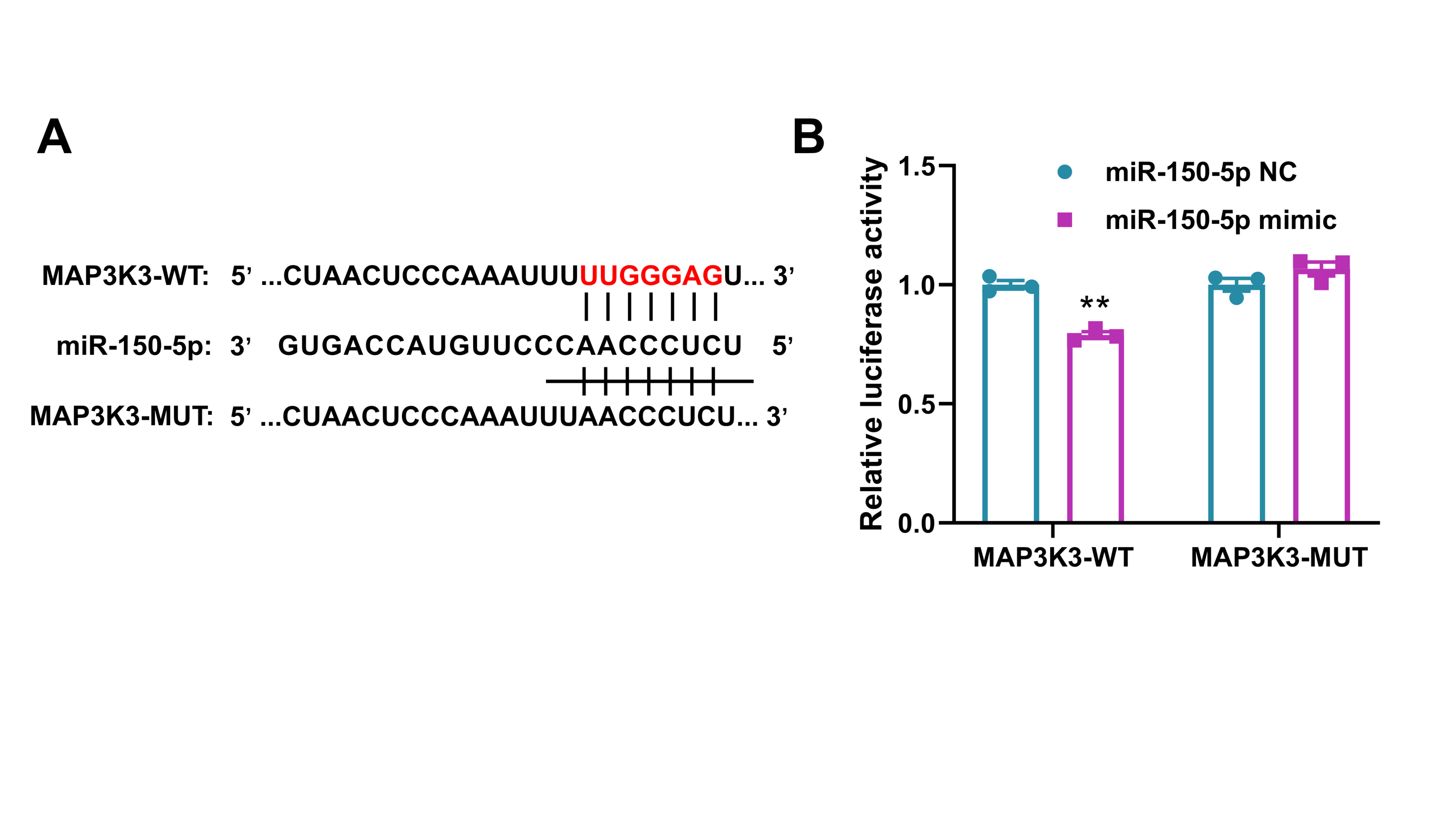

Supplement: Supplementary file 7 — Additional file 7: Fig. S6: Mitogen-activated protein kinase kinase kinase 3 (MAP3K3) was a direct target of miR-150-5p. A Schematic diagram showing miR-150-5p base-pairing with wild-type (WT), but not with mutant (MUT) versions of the 3’ UTR binding site of MAP3K3. B Luciferase activity decreased following co-transfection with miR-150-5p mimic and wild-type 3’-MAP3K3 UTR luciferase plasmid, while no changes were present following co-transfection of the mimic with mutant luciferase plasmid. Luciferase activity in miR-150-5p negative control (NC, comprising scrambled control miRNA) was set at 1.0. Data shown as mean ± SEM. n = 3/group, **p < 0.01. [file 12951_2023_1944_MOESM7_ESM.tif]
